# Marburg Virus Disease outbreaks, mathematical models, and disease parameters: a Systematic Review

**DOI:** 10.1016/S1473-3099(23)00515-7

**Published:** 2023-11-28

**Authors:** Gina Cuomo-Dannenburg, Kelly McCain, Ruth McCabe, H. Juliette T. Unwin, Patrick Doohan, Rebecca K. Nash, Joseph T. Hicks, Kelly Charniga, Cyril Geismar, Ben Lambert, Dariya Nikitin, Janetta Skarp, Jack Wardle, Mara Kont, Sangeeta Bhatia, Natsuko Imai, Sabine van Elsland, Anne Cori, Christian Morgenstern, on behalf of the Pathogen Epidemiology Review Group

**Affiliations:** 1 MRC Centre for Global Infectious Disease Analysis & WHO Collaborating Centre for Infectious Disease Modelling, Jameel Institute, School of Public Health, Imperial College London, UK; 2 Department of Statistics, University of Oxford, UK; 3 Health Protection Research Unit in Emerging and Zoonotic Infections; 4 Health Protection Research Unit in Modelling and Health Economics; 5 Modelling and Economics Unit, UK Health Security Agency, London, UK; 6 College of Engineering, Mathematics and Physical Sciences, University of Exeter, Exeter, UK

**Keywords:** Marburg Virus Disease, MVD, mathematical modelling, epidemiological parameters, systematic review, outbreak analysis

## Abstract

Recent Marburg virus disease (MVD) outbreaks in Equatorial Guinea and Tanzania highlighted the importance of better understanding this highly lethal infectious pathogen. We conducted a systematic review (PROSPERO CRD42023393345), reported according to PRISMA guidelines, of peer-reviewed papers reporting historical outbreaks, modelling studies and epidemiological parameters focused on MVD. We searched PubMed and Web of Science until 31/03/2023. Two reviewers evaluated all titles and abstracts, with consensus-based decision-making. To ensure agreement, 31% (13/42) of studies were double-extracted and a custom-designed quality assessment questionnaire was used for risk of bias assessment. We present detailed information on 478 reported cases and 385 deaths from MVD. Analysis of historical outbreaks and seroprevalence estimates suggests the possibility of undetected MVD outbreaks, asymptomatic transmission and/or cross-reactivity with other pathogens. Only one study presented a mathematical model of MVD transmission. We estimate an unadjusted, pooled total random effect case fatality ratio for MVD of 61.9% (95% CI: 38.8-80.6%, *I*^2^=93%). We identify important epidemiological parameters relating to transmission and natural history for which there are few estimates. This review and the accompanying database provide a comprehensive overview of MVD epidemiology, and identify key knowledge gaps, contributing crucial information for mathematical models to support future MVD epidemic responses.

## Introduction

Infectious disease outbreaks pose a substantial threat to health and well-being globally (1–3). Since the emergence of SARS-CoV-2 at the end of 2019, there have been several other outbreaks of emerging or re-emerging pathogens, including mpox (4), novel hepatitis in children (5), Ebola virus disease (EVD) (6), and Marburg virus disease (MVD) (7,8). These examples demonstrate that the world remains highly vulnerable to infectious disease outbreaks and underscore the importance of developing a better understanding of high-threat pathogens.

In 2018, the World Health Organization (WHO) published a list of nine known infectious diseases for research and development (R&D) prioritisation, due to their epidemic and pandemic potential and the absence of licensed vaccines or therapeutics (9,10). This was updated in 2023 to also include COVID-19 (11). Among the listed pathogens is Marburg virus (MV), a highly lethal infectious *Filoviridae* single-stranded RNA virus of the *Marburgvirus* genus, first described in Germany and Serbia (formerly Yugoslavia) in 1967 (12). Subsequent outbreaks of MVD have primarily occurred in sub-Saharan Africa, including recent outbreaks in Equatorial Guinea and Tanzania in 2023 (7,8)

The host of MV is the fruit bat (*Rousettus aegyptiacus*), with transmission to humans occurring via direct contact with an infected animal host (13,14). Human-to-human transmission has also been observed, primarily occurring in household or healthcare settings with a lack of infection protection and control measures, for example via contact with bodily fluids from an MVD patient (15,16). Phylogenetic analyses have confirmed multiple spillovers from bats to humans (17), but the first known human outbreak was associated with African green monkeys (*Cercopithecus aethiops*) (18). The *Marburgvirus* genus contains two viruses, MV and RAVN, which have a genetic divergence of approximately 20%, with MV having six variants with fewer genomic differences (12). Both viruses are indistinguishable in their clinical presentation, with symptoms comprising, but not limited to, fever, severe headaches and malaise, which can progressively develop into severe hemorrhagic fever, including spontaneous bleeding from one or more orifices (12,18). Although there is a high risk of serious illness upon infection (17), supportive care has been shown to increase the chance of survival in the absence of MVD-specific treatments (18). However, to date, the majority of reported MVD cases have died (12). More comprehensive overviews can be found at (12,18).

Mathematical models of disease transmission and control are a key tool that can be deployed in response to infectious disease outbreaks and are used to guide policy, for example by projecting plausible epidemic trajectories and expected healthcare demand and assessing the potential impact of interventions (19,20). Epidemiological parameters are key inputs to such models, for example governing the rates at which individuals move through disease states. However, gathering information on model structures and appropriate parameter values can be time-consuming and may impede real-time modelling.

To address these issues, we have set out to systematically review the literature relevant to rapid design of dynamic transmission models for high-threat pathogens, here focusing on MVD. We aim to collate available information on MVD outbreaks, modelling studies, and epidemiological parameters related to transmissibility, severity, delays, risk factors, mutation rates and seroprevalence. Our work highlights knowledge gaps and provides a key resource for modelling future outbreaks of MVD or similar (known or unknown) pathogens.

## Methods

PRISMA checklists for this review have been included in Tables D.8 and D.9 in the Supplementary Information.

### Search strategy and study selection

**Figure 1:**
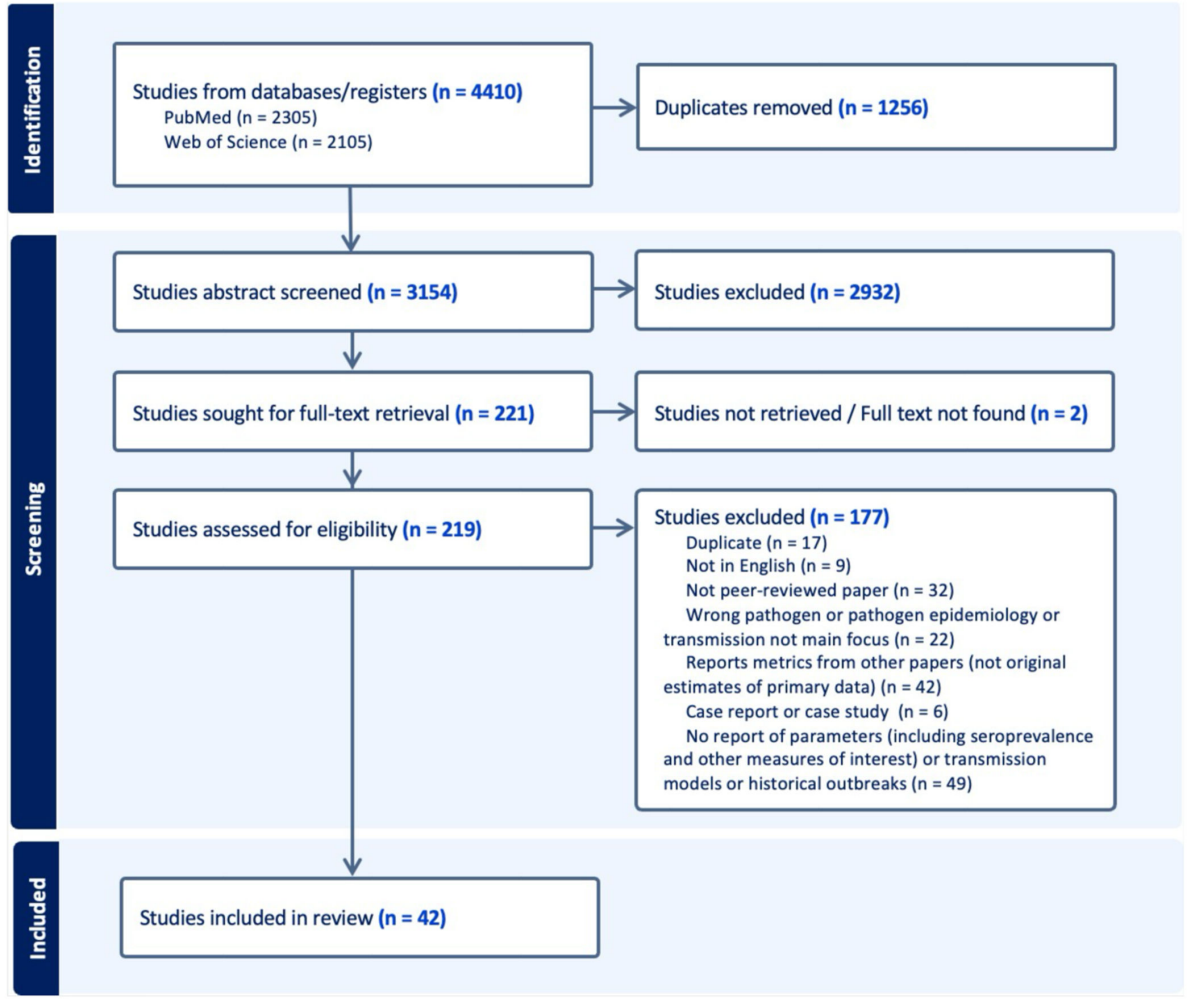
Study selection according to PRISMA guidelines and criteria as described in Table B.2

We searched PubMed and Web of Science for published mathematical transmission models and articles reporting on MVD transmission, evolution, natural history, severity, seroprevalence and size of previous outbreaks, published prior to 31 March 2023 (see the Supplementary Information A.1 for search strategy). Table B.2 presents all inclusion and exclusion criteria. In *Covidence* (21), two independent reviewers screened titles and abstracts then full texts to assess eligibility for data extraction. Covidence does not record reasons for abstract exclusion, but reasons for full-text inclusion and exclusion are recorded in Figure 1. Disagreements were resolved by consensus between reviewers.

### Data extraction

Thirteen reviewers extracted data on article information (publication details, risk of bias), estimated parameters (value, uncertainty range, distribution, context, risk factors), outbreaks (dates, location, case and death numbers) and models (model type and structure, interventions modelled, transmission routes, assumptions) from the included studies into a Microsoft Access database (Version 2305), with one reviewer per paper. Risk of bias was assessed using a seven-question form addressing the quality and reliability of the methodology, assumptions, and data. For a randomly selected 30% (13/43) of papers, extraction was performed by two independent reviewers. Consensus on discordant results was established before single reviewer data extraction commenced. More details are provided in the Supplementary Information A.

We collated information only from outbreaks that were reported to be complete.

We extracted parameter values, units, uncertainty intervals (capturing the precision of estimates), and ranges (capturing heterogeneity in estimates across different population groups, time or space) for all parameters except risk factors. Study context was also recorded, when reported. We extracted risk factors investigated in the studies and whether they were statistically significant and/or adjusted. We chose not to extract odds ratio estimates because varying stratifications and reference groups complicates comparison across studies.

Information extracted about previous outbreaks, namely cases and deaths, was further used to generate estimates of the case fatality ratio (CFR).

Full details on data extraction, including descriptions of variables and predefined options for categorical variables, can be found in the Supplementary Information Tables B.2 – B.5.

### R package

We designed an R package, *epireview*, where all curated data on epidemiological parameters, models and outbreaks are publicly available (22). A dedicated vignette explains how independent contributors may add information to the package, so that it provides a live view of the latest knowledge on MVD. More details can be found in the Supplementary Information C.

### Data analysis

We use descriptive tables and figures to present the collated data. Unless otherwise specified, uncertainty intervals in tables and figures (e.g. 95% confidence (CI) or credible intervals (CrI)) were extracted from the papers or computed from reported central estimates and standard errors (SI A.3).

In the following, an “unadjusted CFR estimate” refers to an estimate where raw deaths are divided by raw cases, with no weighting or controlling for other variables or cases with unknown outcome.

We conducted two meta-analyses for the case fatality ratio (CFR), using 1) CFR estimates extracted from the studies, and 2) unadjusted CFRs that we computed from extracted outbreak data. Comparison between the two sets of CFR estimates enabled assessment of any bias due to outbreaks for which there was no or multiple reported CFR estimates in the literature. For this analysis, we defined an ‘outbreak’ as one or more cases identified in the same country and within the same date ranges. This included single cases, often related to zoonotic spillover or importation events, and large outbreaks. We ensured that each case was counted only once: if multiple studies reported the same outbreak, we chose the study covering the longest time period. We estimated exact 95% binomial confidence intervals on individual outbreak estimates.

Meta-analyses were performed using the meta R package (23) providing a *total common effect* and a *total random effect* pooled CFR estimate with 95% CI and statistics on heterogeneity in CFR across studies. Further details and references are provided in the Supplementary Information A.4.

Overall quality assessment scores were calculated as a mean of the responses to the seven questions, excluding non-applicable questions (that is, if the quality assessment question was not applicable to a study, it did not contribute to the quality assessment score). A local polynomial regression fit using the *R* function *loess* was used to analyse trends in quality assessment scores by publication year. Although we excluded systematic reviews from our search, we used those listed in Supplement E to ensure that no outbreaks were missed and that parameter estimates were within the previously reported ranges. Analyses were conducted using *R* (version 4.2.2).

## Results

The search returned 4410 studies (2305 from PubMed and 2105 from Web of Science) from which we removed 1256 duplicates. Of the remaining 3154 studies for which we screened abstracts, 221 were kept for full-text review. Studies were further excluded for various reasons, including not reporting any parameter or original parameter estimates, not being peer-reviewed, being duplicated, and being in a non-English language. 42 studies were included for data extraction. The PRISMA flowchart further describes the study selection (Figure 1).

We collated evidence from 13 studies reporting 23 observed MVD outbreaks. Based on timings and locations reported in the studies, we identified seven distinct outbreaks (Table 1). This included the first identified outbreak in Marburg, Germany, and the former Federal People’s Republic of Yugoslavia from which MVD was identified and named; an outbreak in the Democratic Republic of the Congo (DRC) from 1998 - 2000; a series of cases from Johannesburg, South Africa in early 1975 (linked to prior travel to Zimbabwe); three outbreaks in Uganda; and an outbreak in Angola in 2004 - 2005. In addition, we noted the reporting of individual MVD cases in Kenya in 1980 and 1987 (likely caused by animal exposure); in the Russian Federation in 1988 and 1990 (both linked to a laboratory worker in a research facility); and in the Netherlands and the United States of America in 2008, both linked to the 2007 Ugandan outbreak. At the time of the literature search, there were no peer-reviewed studies on the 2023 MVD outbreaks in Equatorial Guinea and Tanzania. We include both outbreaks in (Table 1) based on WHO reported numbers after the end of the outbreak was declared. These numbers are not included in the epireview database, which could be updated in the future as and when peer-reviewed papers are available.

The only transmission modelling study of MVD was by Ajelli et al, 2012. The authors used a stochastic, individual- based, SEIR model to examine the impact of behaviour change interventions on MVD cases and deaths (24). Transmission in the model occurred via direct, non-sexual human contact, assuming homogeneous mixing; transmission rates were heterogeneous over time, with temporal changes in viral load and hence transmissibility; susceptibility was assumed to be age-dependent, and the latent and incubation periods were assumed to coincide (24). The potential impact of quarantine was simulated, though was not explicitly based on real-world data. As detailed below, the authors provided estimates of generation time and basic reproduction number (24).

Overall, we extracted 71 epidemiological parameter estimates: see overview in Figure B.1 in the Supplementary Information and parameter definitions and details of the extraction process in the accompanying R package *epireview* (22). Seroprevalence estimates were the most frequently reported in the literature, followed by delays and severity. Two studies reported on transmission parameters (e.g., attack rates and reproduction numbers), and four provided estimates of evolutionary mutation rates. We also extracted reported risk factors for different outcomes, namely infection, severe disease, seropositivity, recovery, and death.

**Figure 3:**
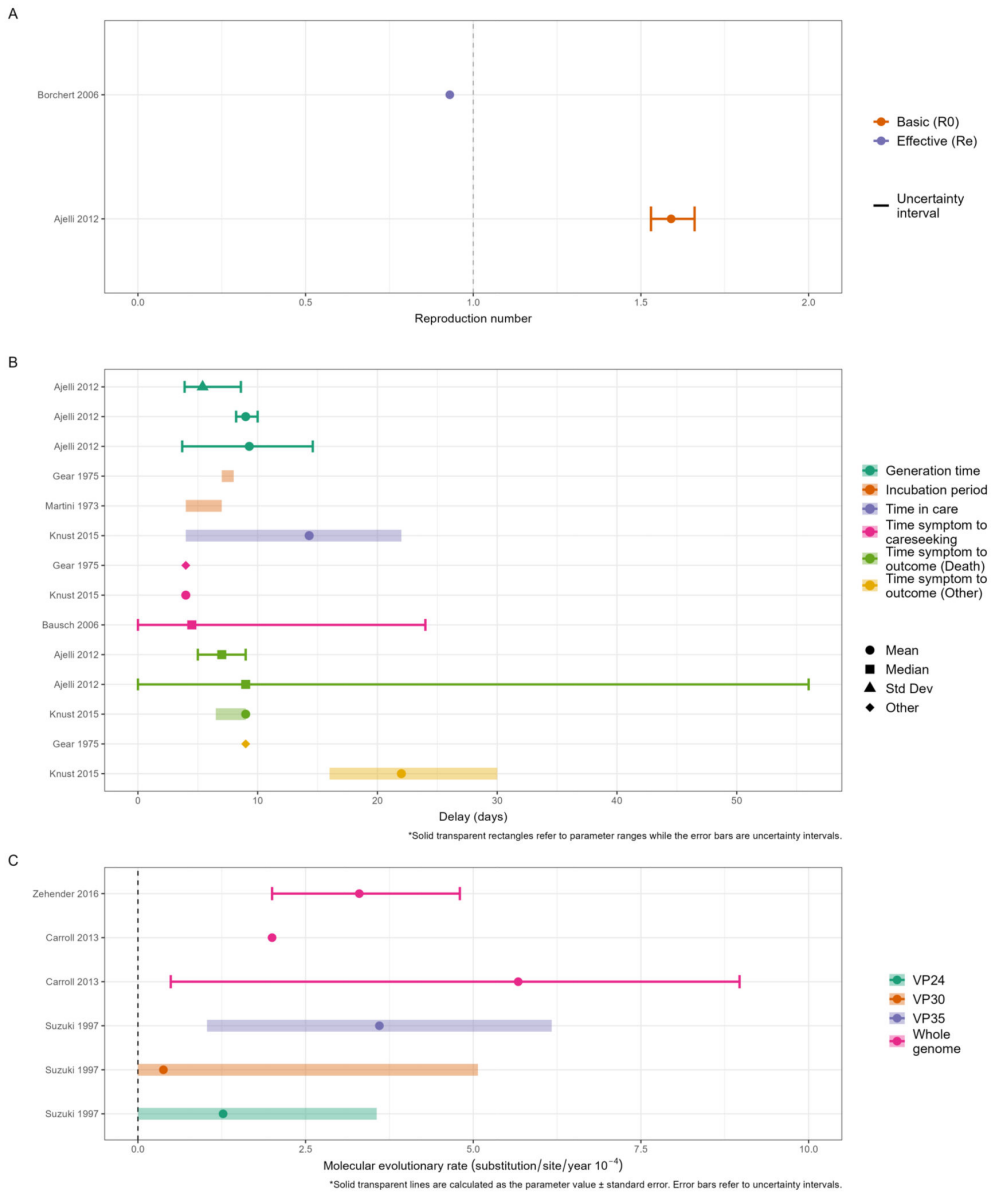
Overview of the reproduction numbers, delays and evolutionary rate estimates from the included studies of MVD. Solid lines represent uncertainty intervals and ribbons indicate a parameter range (e.g. across different populations or over time). (A) Estimates of the reproduction number. The blue and red points correspond to estimates of the effective reproduction number (*R_e_*) and basic reproduction number (*R*_0_) respectively, with associated uncertainty shown by the solid lines where available. The dashed vertical line presents the threshold for epidemic growth. (B) Delay parameters, stratified into five categories: Generation Time, Incubation Period, Time in Care, Time from Symptom to Careseeking and Time from Symptom to Outcome as indicated by different colours. (C) Evolu- tionary rates. Colours indicate different genome types; points represent central estimates. Solid lines represent an uncertainty interval associated with the point estimate while ribbons indicate a parameter value +/- standard error with a minimum set to zero.

Reproduction number estimates were reported in two studies (24,25). Ajelli et al. used a mathematical model (see above), to estimate the basic reproduction number, *R*_0_, for the 2005 Angola outbreak. They found that *R*_0_ = 1.59 (95% CI: 1.53–1.66), suggesting that in the absence of mitigation efforts, the virus would be expected to propagate in a similar population (24). They also provided the only estimate of doubling time, at 12.4 days (95% CI: 11.3–13.6 days) (24). Borchert et al. estimated the effective reproduction number, *R_e_*, based on secondary attack rates derived from seroprevalence in contacts of confirmed cases in DRC in 2002 (25). This study also provided the only estimate of attack rate, at 21% (Figure 3).

Six CFR estimates were reported, corresponding to the outbreaks in Angola in 2005 (24), DRC in 1999 (17), the original 1968 outbreak in Germany and Yugoslavia (26) and three estimates from the 2012 Uganda outbreak (27,28) (Figure B.2A SI). Pooling these estimates gave a total common effect CFR of 80.6% (95% CI: 77.3-83.6%, *I*^2^=93%) and a total random effect CFR of 61.9% (95% CI: 38.8-80.6%, *I*^2^=93%). We additionally estimated an unadjusted, pooled CFR using the extracted historical outbreak data (Figure B.2B SI), combining data from 467 confirmed cases and 11 suspected cases across 13 distinct outbreaks with 385 reported deaths. The pooled common effect CFR estimate from the extracted outbreak data was 80.5% (95% CI: 76.7-83.8%, *I*^2^=82%) and the pooled random effect CFR 63.8% (95% CI: 41.6-81.3%, *I*^2^=82%), both highly consistent with the previous estimates based on CFR parameters reported in the literature.

We collated estimates of the generation time, incubation period, time in care, and time from symptom onset to careseeking, death or other outcomes as summarised in Figure 3 and Table B.6 in the Supplementary Information. The two generation time estimates were based on viral load data from non-human primates under two distinct assumptions, namely that infectiousness is directly proportional to viral load, and alternatively assuming that probability of death is directly proportional to viral load (24,29). This study also estimated the time from symptom onset to death using additional assumptions about these relationships (24). The sole estimate of time in care was a median of 14.3 days (range 4 - 22 days) that 6 survivors of the 2012 Uganda outbreak spent in care, with a median duration in isolation of 22 days (16 - 30 days) (27). The two incubation period estimates came from studies from the 1970s only reporting ranges with little overlap (26,30) (Figure 3). Central estimates of time from symptom onset to careseeking across the 1975 South Africa, 1998 DRC, and 2012 Uganda outbreaks were consistently under 5 days, although Bausch et al. showed a large range of delays from symptoms to seeking medical care (17,27,30) for the 1998 DRC outbreak.

We extracted 15 risk factors for MVD infection and seropositivity from 4 studies which are presented in Table B.7 in the Supplementary Information (13,17,27,31). Having had contact with confirmed MVD cases, including through working in funeral and burial services, was a statistically significant risk factor for infection. The ‘other’ classification encompassed a wide range of factors, such as prevalence of infection in the host reservoir, subsistence activities and previous invasive medical treatment, and as such are not directly comparable, although some constituted statistically significant risk factors (13,27,31). Sex was not significantly associated with MVD infection (27). Although similar risk factors were explored to assess impact on seropositivity, the only significant risk identified for this outcome was known hospitalisation with MVD.

Three studies reported molecular evolutionary rates of MV, two estimated using whole genome sequencing (32,33) and one based on individual genes (34). The three evolutionary rate estimates from whole genomes are largely consistent with one another, whilst those based on individual genes tended to be lower (Figure 3C).

Twenty-one studies contained seroprevalence estimates across a 38-year period from 1980 - 2018 in 15 predominantly Sub-Saharan African countries (25,26,31,35–52) (Table 2). Presence of antibodies was assessed using a range of assays: Indirect Fluorescent Antibody assay (IFA) (6 studies (31,36,40,42,49,53)); Hemagglutination Inhibition Assay (HAI/HI) (1 study (47)); Immunoglobulin G (IgG) (7 studies (25,35,37,46,51,52,54)); Immunoglobulin M (IgM) (2 studies (43,44)); the remaining studies did not specify the assay used (3 studies [35, 22, 41]). IgG and IgM were used for all recent studies (from 1995 onwards), highlighting recent developments in serology and the retiring of assays testing for IFA and HAI/HI. The studies included in this review demonstrated low levels of antibodies in surveyed populations, with approximately one third of studies reporting a seroprevalence of 0% (26,36,39,41,46,50,52). Among studies with estimates above zero, seroprevalence ranged from 0.5% in the Republic of the Congo in 2011 (54), to 2.1% in healthcare workers in DRC in 2001 - 2002 (52), to 4.5% in Uganda in 1984 (47). Overall, the evidence gathered here indicates high susceptibility to MVD among populations in the surveyed regions, including Tanzania, where one of the subsequent 2023 MVD outbreaks occurred (44). However, these seroprevalence estimates must be interpreted in the context of the very small sample sizes of most studies.

The results of the quality assessment are summarised in (Figure B.3A SI). The number of non-applicable answers are driven by more descriptive studies, such as seroprevalence studies, which did not use a model or statistical analysis. Papers on transmission parameters had on average the highest quality assessment scores (reproduction number paper score = 0.80, other transmission parameters papers score = 0.87, we note the small number of papers in this category) and papers on seroprevalence the lowest score of 0.48. Scores improved over time (Figure B.3B in SI) which may also explain the difference in quality assessment score between types of parameters, as seroprevalence papers tended to be published much earlier than other study types.

## Discussion

This systematic review presents a comprehensive set of mathematical models, outbreaks, and epidemiological parameters of MVD. Historical outbreaks and case reports in the peer-reviewed literature for MVD were rare and small in size, relative to many other pathogens, including other viral hemorrhagic fevers such as Ebola virus disease (EVD), with only 7 notable outbreaks reported (Table 1). Only two outbreaks had over 100 confirmed cases (DRC 1998: 154 cases, Angola 2005: 254 cases), with the remainder reporting 31 cases or fewer. For most parameters, we were only able to obtain a small number of estimates, a substantial number of which were only reported as point estimates with no uncertainty. Seroprevalence of MVD was the metric most widely reported across a large number of locations in Sub-Saharan Africa (Table 2) and indicates that seroprevalence is generally low. However, serosurveys suggest that some past MVD outbreaks may have gone undetected. Reported seroprevalence in the Central African Republic (CAR) is relatively high (3.2%, range among subgroups: 1.0- 7.4%) despite having no recorded MVD outbreaks, although these results may stem from cross-reactivity or low assay specificity. Seroprevalence estimates of MVD and EVD are often reported together, with estimates for MVD consistently lower than for EVD, e.g. (35,36,44,54).

A basic reproduction number of 1.59 (95% CI: 1.53-1.66) was estimated for the largest known outbreak to date in Angola (24). However, Borchert et al. (25) estimated an effective reproduction number, *R_e_*, of 0.93 for the 1998 DRC outbreak after the introduction of public health and social measures (PHSM), suggesting that such interventions can effectively mitigate MVD transmission.

The pooled CFR estimates for MVD provide several key insights. The pooled random effects CFR of 61.9% (95% CI: 38.8-80.6%, *I*^2^=93%) highlights the heterogeneity in CFR across outbreaks. In comparison, the pooled common effect CFR of 80.6% (95% CI: 77.3-83.6%, *I*^2^=93%) is skewed towards the two large outbreaks in Angola and Uganda, which had very high CFRs, and presents a possibly misleadingly narrow uncertainty interval but highlights that MVD outbreaks with higher transmissibility may also be associated with higher severity. The results from the meta-analyses of reported CFR parameters and computed, unadjusted CFR from outbreak data are consistent, and our estimates are in line with a previous systematic review (55). All CFR estimates, irrespective of the method, are extremely high, implying very high costs of human life in the endemic countries, so far all located in Sub-Saharan Africa. Low seroprevalence estimates in these regions, combined with high fatality and a basic reproduction number above one, clearly demonstrate the pandemic potential of MVD.

The gaps in knowledge of MVD are substantial. Although we found some epidemiological estimates, several of them are from the previous century and based on poor-quality data; for example, most estimates of the CFR for MVD reported in the literature were unadjusted estimates. Crucial model inputs, such as the generation time, were estimated from primate studies and would benefit from confirmation from human outbreak data. MV evolutionary rates were also imperfectly characterised, across only a handful of sometimes very dated studies with small sample sizes and some methodological issues, including not accounting for synonymous mutations. The reported substitution rates are substantially lower than those reported for Ebola (56). Although such comparison should be interpreted with the caveats above in mind, this points to MV evolving approximately 3 times more slowly than Ebola. With a shorter mean generation time (approximately half that of Ebola), these results suggest that very little pathogen genetic diversity between MVD cases is to be expected, and hence genomic data might be of limited value in inferring transmission trends in future MVD epidemics (56). However, better characterisation of MVD evolution should be prioritised on the research agenda.

Recent outbreaks of MVD in Equatorial Guinea and Tanzania were controlled through basic measures such as *Infection Prevention and Control* and *Risk Communication and Community Engagement* (57). WHO declared the end of the Equatorial Guinea outbreak on 8 June 2023 (58) (17 laboratory-confirmed cases, 12 deaths, and a further 23 probable cases, all of whom died) and the Ministry of Health of the United Republic of Tanzania confirmed the end of the first MVD outbreak in Tanzania on 2 June 2023 (59) (8 laboratory-confirmed cases, 1 probable case and 6 deaths). These are severe and traumatic events for the communities impacted but are also opportunities to gather higher-quality data. In particular, careful collection of patient information, documentation of disease progression and regular follow-ups post-infection would enable the research community to better characterise epidemiological delays and risk factors for infection and death.

The collection of parameters presented here, a synthesis of peer-reviewed information up to March 2023, will enable researchers to construct and parameterise simple epidemiological models for MVD. Our accompanying R package *epireview* (22) will facilitate this process and ensure that information from studies beyond March 2023 can be added to the package, thereby offering a continuously updated repository of parameter estimates. The importance of this work is underlined by the scarcity of published MVD mathematical models, which contrasts with the abundance of published models describing EVD (60).

Improved knowledge of parameters will enable more modelling analyses to explore the potential impact of interventions such as PHSM, as has been done for EVD (61). Although there is no vaccine approved for MVD, phase 1 clinical trials have shown promising results (62). Mathematical models could support the design of vaccination strategies, as they did for EVD (63).

This review was challenging as it contained a wide variety of studies and parameters for which we could not find a unique pre-existing, validated quality assessment tool. We therefore constructed a scoring system tailored specifically to the broad range of information we were collating to assess the validity of the methods, assumptions, and data,. We observed an improvement in paper quality over time, which we attribute to increasing transparency in models, assumptions and data (including publication of data and code), which enables reproducibility of research. Our findings are constrained by our restriction to peer-reviewed articles in English, a particularly important limitation given that many of the countries reported to have experienced MVD outbreaks are not English speaking. Extending this work to include non-English language articles and non-peer reviewed work to avoid language bias and provide a more comprehensive and generalisable picture is an interesting avenue but would be challenging. Lowering the hurdles for mathematical epidemic model design is important to enable timely generation of evidence that can support epidemic response to future outbreaks. Here, we provide a comprehensive summary of published mathematical models, outbreaks, and epidemiological parameters of MVD. Our work summarises existing information on MVD dynamics and highlights key knowledge gaps which would benefit from further elucidation. Future research should similarly review the available evidence on past outbreaks, mathematical models and epidemiological parameters for other high-threat pathogens such as those on the WHO blueprint priority list (9). Alongside this paper, we publish the database of extracted MVD models, parameters and outbreaks, thus enabling future additions as more information becomes available from future studies, on MVD or other pathogens. Information is synthesised in the R package epireview [18], which also includes functionalities to visualise the latest information, thereby providing a continuously up-to-date picture of MVD epidemiological knowledge. This tool should further enhance global epidemic modelling preparedness.

## Supplementary Material

SI

## Figures and Tables

**Figure 2:**
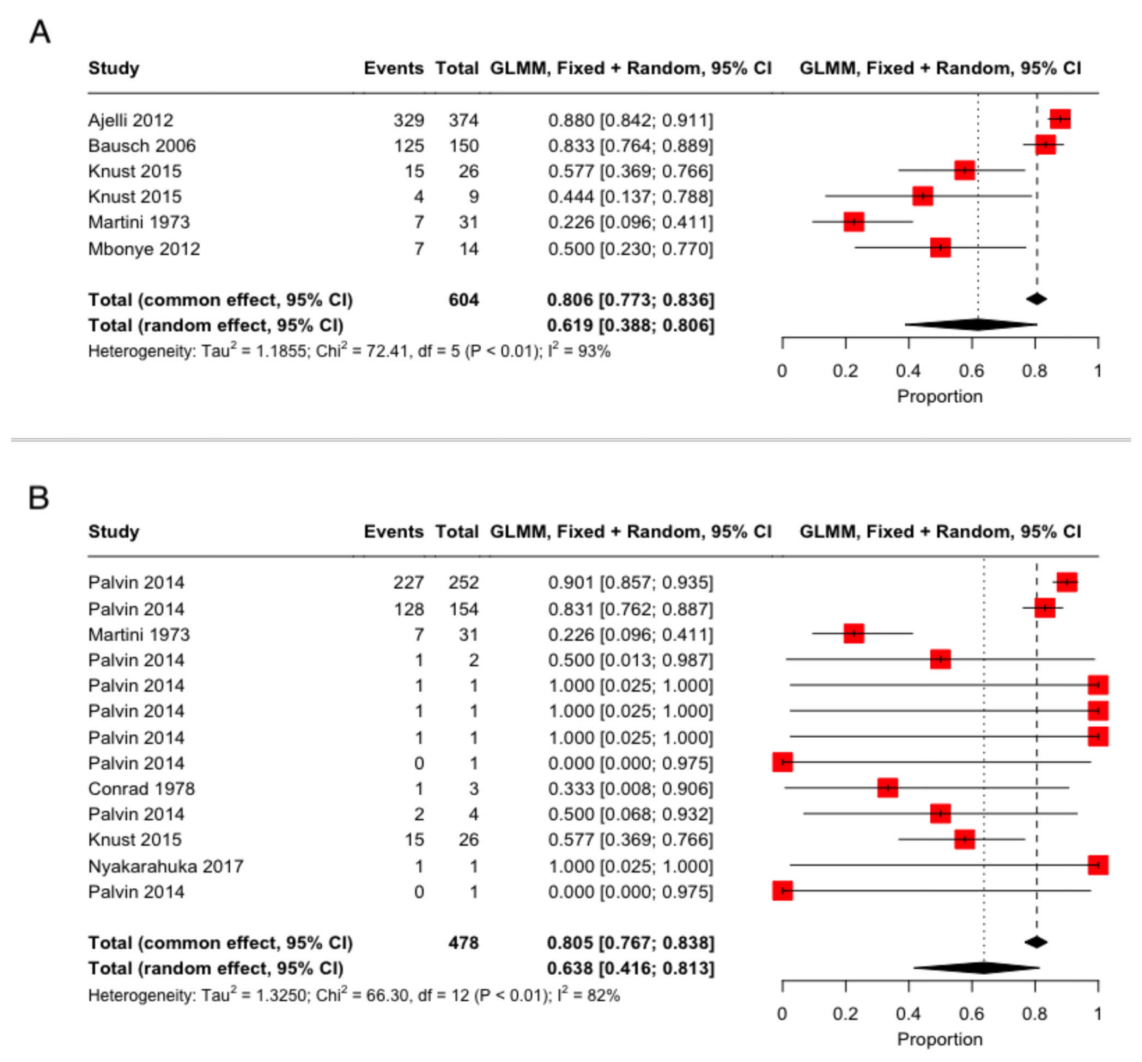
Case Fatality Ratio (CFR) meta-analyses, using logit-transformed proportions and a generalized linear mixed-effects model (GLMM) (full details in SI A.4). The forest plot displays studies included in each meta-analysis: the red squares indicate study weight, and for each study, a 95% binomial confidence interval is provided. To summarize, we display as black diamonds the *total common effects*, where all data are effectively pooled and assumed to come from a single data-generating process with one common CFR and *total random effect* estimates, which allow the CFR to vary by study and accordingly give different weights to each study when determining an overall estimate (23). (A) CFR estimates reported in the included studies. (B) CFR estimated from extracted outbreak data, including only one observation per outbreak using the study with the longest duration of the outbreak reported ensuring no case is double counted.

**Table 1:** Overview of MVD outbreaks i.e. location, timing, and size, as reported in the studies included in this review. We report in bold the country and outbreak year, the location refers to the place of the actual outbreak in the country if known. Blank cells correspond to information which we were unable to find in or extract from the literature. *: The 2023 outbreaks in Equatorial Guinea and Tanzania are captured from WHO announcements after the end of the outbreaks was declared. This data is not from peer-reviewed articles and not captured in the epireview database. (§) Unique outbreaks (478 reported cases [confirmed and suspected] and 385 deaths).

Location	Article	Start	End	Deaths	Cases				Confirmation
					Confirmed	Suspected	Asymptomatic	Severe/hospitalised	Method
***Germany, Yugoslavia - 1968***										
Marburg, Frankfurt am Main	Albarino 2013		18 Aug 1968	13 Nov 1968	5	23		1		Symptoms
Marburg, Frankfurt am Main, Belgrade	Martini 1973	(§)	Aug 1968	Nov 1968	7	31		1		Symptoms
	Palvin 2014		1967		7	31				
***South Africa, Zimbabwe - 1975***										
Johannesburg	Conrad 1978	(§)	12 Feb 1975		1	3			1	Molecular
***Kenya - 1980***										
	Palvin 2014	(§)	1980		1	2				
***Kenya - 1987***										
	Palvin 2014	(§)	1987		1	1				
***Russian Federation - 1988***										
	Palvin 2014	(§)	1988		1	1				
***Russian Federation - 1990***										
	Palvin 2014	(§)	1990		0	1				
***Democratic Republic of the Congo - 1998***										
Durba and Watsa	Borchert 2002		Oct 1998	May 1999	61	73			0	Symptoms
Durba and Watsa	Bausch 2006		Oct 1998	Sep 2000	125	48	106		0	Molecular
Durba and Watsa	Borchert 2006		Oct 1998	Aug 2000		76	33	0		Molecular
	Palvin 2014	(§)	1998	2000	128	154				
***Angola - 2005***										
	Carroll 2013		2005	2005	227	252			0	
	Palvin 2014	(§)	2004	2005	227	252				
Uige Province	Towner 2006		Oct 2004	Jul 2005	227	252			0	
***Uganda - 2007***										
Kamwenge and Ibanda	Adjemian 2011		10 Jun 2007	14 Sep 2007	1	4			0	Molecular
	Palvin 2014	(§)	2007		2	4				
***Netherlands - 2008***										
	Palvin 2014	(§)	2008		1	1				
***United States – 2008***										
Colorado	Palvin 2014	(§)	2008		0	1				
***Uganda - 2012***								
Kabale; Ibanda; Mbarara; Kampala	Albarino 2013	18 Oct 2012	7 Nov 2012	4	15			Molecular
Ibanda, Kabale and Kamwenge Districts	Knust 2015 (§)	Jul 2012	10 Nov 2012	15	15	11		Molecular
Ibanda and Kabale	Mbonye 2012	Sep 2012	13 Nov 2012	7	9	5		Molecular
***Uganda - 2014***								
Kampala	Nyakarahuka 2017 (§)	17 Sep 2014	28 Sep 2014	1	1		1	Molecular
Equatorial Guinea - 2023*								
	WHO 2023	13 Feb 2023	8 Jun 2023	35	17	23		Molecular
Tanzania – 2023*								
Bukoba district (Kagera region)	WHO 2023	21 Mar 2023	2 Jun 2023	6	8	1		Molecular

**Table 2:** Overview of seroprevalence estimates for MVD as reported in the included studies. Estimates were primarily reported as percentages. Associated uncertainty and sample sizes are provided where these were reported. Where available, additional information regarding the location and timing of the estimates, the antibody being tested for, the target population, the timing in relation to any ongoing outbreak and the availability of disaggregated data is also summarised.

Article	Survey year	Parameter type*	Seroprevalence (%)	Uncertainty (%)	Uncertainty type	Number Seropositive	Sample size	Population Group	Timing of survey	Disaggregated data available
***Central African Republic***
Gonzalez 2000	Nov 1995	IgG	2.40%	0 - 5.6	Range	33	1340		
Johnson 1993		IFA				3	427	Outdoor workers	
Johnson 1993		IFA	3.20%	1.0 - 7.4	Range	137	4295	General population		Age, Region, Sex
***Republic of the Congo***
Moyen 2015	03Mar-07Jul 2011	IgG	0.50%						Pre outbreak	
***Democratic Republic of the Congo***
Bausch 2003	May 1999	IgG	2.00%			15	912		Mid outbreak
Borchert 2005		IFA	0.00%	0 - 1.2	Range	0	300		Post outbreak
Borchert 2006		IgG	1.65%	0.2 - 5.8	CI95%			Household contacts of survivors	Post outbreak
Borchert 2007	2001-2002	IgG	2.10%					Healthcare workers	Post outbreak
***Gabon***									
Ivanoff 1982	Feb-Mar 1980	IFA	0.00%			0	197		
Ivanoff 1982	Feb-Mar 1980	IFA	0.00%			0	28	Pregnant women	
***Germany***									
Becker 1992		IgG	2.60%					Other	
***Guinea, Liberia, Sierra Leone***
O’Hearn 2016	2007-2014	IgG	10.70%	71	663	Other
***Kenya***									
Johnson 1983a	1980-1981	IFA		8	1899					Region
Johnson 1983b		IFA	0.00%	0	741	General population		Post outbreak	
Martini 1973		Unspecified		0	79	Other		Post outbreak	
Smith 1982		Unspecified		2	186	Persons under investigation		Post outbreak	
Smith 1982		Unspecified		3	100	Healthcare workers		Post outbreak	
Smith 1982		Unspecified		0	224	General population		Post outbreak	
Smith 1982		Unspecified		0	63	Other		Post outbreak	
Smith 1982		Unspecified		0	79	Other		Post outbreak	
Smith 1982		Unspecified		0	44	Outdoor workers		Post outbreak	
***Liberia***								
Van der Waals 1986	1981-1982	IFA	1.30%	3	225	Other		Other		Other, Region
***Madagascar***								
Mathiot 1989		Unspecified	0.00%	0	381			Other		Region
***Nigeria***								
Tomori 1988		IFA	1.70%	29	1677	General population		
***Sierra Leone***										
Schoepp 2014	2006-2008	IgM	3.60%			Persons under investigation		Other	
***Tanzania***								
Rugarabamu 2022	Jun-Nov 2018	IgM	0.30%	1	308			Other		Region
***Uganda***								
Evans 2018	Mar-Jul 2013	IgG		0	331			Other	
Rodhain 1989	May-May 1984	HAI/HI	4.50%	6	132			
***Cameroon, Central African Republic, Chad, Republic of the Congo, Equatorial Guinea, Gabon***
Gonzalez 1989	1985-1987	Unspecified	0.39%	20	5070	General population				Region

* HAI/HI: Hemagglutination Inhibition Assay; IFA: Indirect Fluorescent Antibody assay; IgG: Immunoglobulin G; IgM: Immunoglobulin M; Unspecified: Unspecified assay.

## Data Availability

https://github.com/mrc-ide/epireview/tree/main/data

## Key Points

- We provide an overview of all outbreaks of MVD to 31/03/2023 consisting of 478 reported cases and 385 deaths.
- We estimate an unadjusted, pooled total random effect case fatality ratio for MVD of 61.9% (95% CI: 38.8-80.6%, *I*^2^=93%).
- We identify important epidemiological parameters relating to MVD transmission and natural history that are poorly characterised in the literature.
- The extensive collection of knowledge gathered here will be crucial in developing mathematical models for use in the early stages of future outbreaks of MVD.
- All data are published alongside this article in the R package epireview, with functionality to easily update the database as new data become available.

## Code availability

https://github.com/mrc-ide/epireview
