# Supplementary material for: Marburg Virus Disease outbreaks, mathematical models, and disease parameters: a Systematic Review
Source: Lancet Infect Dis. Author manuscript; Available in PMC 2024 May 1. (PMC7615873; doi:10.1016/S1473-3099(23)00515-7)
Supplement: SI [file EMS192509-supplement-SI.pdf]

Supplementary Information

Marburg Virus Disease outbreaks, mathematical models, and disease parameters: a Systematic Review

Gina Cuomo-Dannenburg[1,*], Kelly McCain[1,*], Ruth McCabe[1,2,3,*], H. Juliette T. Unwin[1], Patrick Doohan[1], Rebecca K. Nash[1], Joseph T. Hicks[1], Kelly Charniga[1], Cyril Geismar[1,4], Ben Lambert[6], Dariya Nikitin[1], Janetta Skarp[1], Jack Wardle[1], Mara Kont[1], Sangeeta Bhatia[1,4,5], Natsuko Imai[1], Sabine van Elsland[1,+], Anne Cori[1,4,+], and Christian Morgenstern[1,*,#], on behalf of the *Pathogen Epidemiology Review Group*[m]

[1]MRC Centre for Global Infectious Disease Analysis & WHO Collaborating Centre for Infectious Disease Modelling, Jameel Institute, School of Public Health, Imperial College London, UK

[2]Department of Statistics, University of Oxford, UK

[3]Health Protection Research Unit in Emerging and Zoonotic Infections

[4]Health Protection Research Unit in Modelling and Health Economics

[5]Modelling and Economics Unit, UK Health Security Agency, London, UK

[6]College of Engineering, Mathematics and Physical Sciences, University of Exeter, Exeter, UK

[*,+]Contributed equally

[m] Membership of group authorship is listed in the Supplementary Information

[#] Corresponding author: c.morgenstern@imperial.ac.uk

July 2023

Contents

A Additional Information on Methods **2**
- A.1 Study selection . . . 2
- A.2 Data Extraction . . . 2
- A.3 Analysis . . . 3
- A.4 Meta-analysis methods . . . 3
- A.5 Quality Assessment . . . 3

B Additional Figures & Tables **4**

C epireview **12**

D PRISMA 2020 Checklists **13**

E Systematic Reviews **16**

F Excluded Studies **17**

G Pathogen Epidemiology Review Group (PERG) membership **33**

A Additional Information on Methods

A.1 Study selection

Original research papers in English were included if reporting on MVD transmission, evolution, natural history, severity, seroprevalence, size of previous outbreaks or published mathematical transmission models. Non-peer reviewed literature was excluded. Papers identified in the search were imported into *Covidence*, a software program used to manage systematic reviews. From a team of seven reviewers, two independent reviewers first screened titles and abstracts then full texts to assess eligibility for data extraction. Disagreements in eligibility determination were resolved by consensus between the two independent reviewers.

Search terms: `Marburg virus AND ((transmissi* OR epidemiolog*) OR (model* NOT imag*) OR (severity OR \case fatality ratio*" OR CFR OR \case fatality rate*" OR \mortality rate*" OR \attack rate*") OR (\infectious period*" OR \serial interval*" OR \incubation period*" OR \generation time*" OR \generation interval*" OR \latent period*" OR latency) OR (heterogeneit* OR superspread* OR \super spread*" OR super-spread* OR overdispersion OR overdispersed OR over-dispersion OR over-dispersed OR \over dispersion" OR \over dispersed") OR (infectivity OR infectiousness OR \growth rate*" OR \reproduction number*" OR \reproductive number*" OR R0 OR \reproduction ratio*" OR \reproductive rate*") OR (\pre-existing immunity" OR serological OR serology OR serosurvey*) OR (evolution* OR mutation* OR substitution*) OR (outbreak* OR cluster* OR epidemic*) OR (\risk factor*"))`

A.2 Data Extraction

Outbreaks

Possible methods of case confirmation included rapid diagnostic tests (RDTs) or polymerase chain reaction (PCR) tests.

Models

Details of models of disease transmission were extracted, including model type, whether it modelled deterministic or stochastic processes, whether the model was theoretical or fitted to data, availability of model code, and in the case of a compartmental model, model subclassification (e.g., SIR, SEIR). Finally, we extracted information on transmission routes modelled, underlying model assumptions, and interventions included within the model.

Parameters

Survey context of parameter values included survey location and dates, sample size, basic demographic information, and timing of the survey in relation to reported outbreaks. For each parameter, we extracted all available information, including types of values (e.g., mean, standard deviation, median), uncertainty intervals (capturing the precision of estimates), and ranges (if when multiple estimates were obtained from different populations or using different methods).

We recorded the methods used for reproduction number estimation (e.g. renewal equations, empirical methods, compartmental models). For case fatality ratios, we extracted whether the estimation approach accounted for cases with unknown final status or not. For case fatality ratios and seroprevalence, we additionally recorded numerators and denominators where available. For genomic data, we noted the gene studied if specified and if the sequence data were available.

Outcome (e.g. infection or death), the risk factor for that outcome (e.g. age, sex or occupation), the type of occupation if specified, and whether the risk factor(s) estimates were statistically significant and/or adjusted. We chose not to extract odds ratio estimates because studies may have used different stratifications or reference groups, making it challenging to compare values across studies. The information we extract offers an overview of risk factors explored across studies that may affect the risk of infection and death, which may be useful to consider when designing MVD transmission models.

A.3 Analysis

We presented the de-duplicated start and end dates, deaths, confirmed, suspected, asymptomatic, and severe/hospitalised cases, and method of case confirmation in Table B.3.

Confidence intervals for the unadjusted CFR estimates, as shown in Figure B.2, were computed as $(\hat{CFR} \pm 1.96 \times \sqrt{\frac{\hat{CFR} \times (1-\hat{CFR})}{n}}$, where n denotes the number of cases (e.g. the denominator used to derive the CFR estimate).

The mean generation time of 9.15 days is computed as the simple average of the two extracted generation time parameters from Ajelli et al [1] of 9 days and 9.3 days.

A.4 Meta-analysis methods

We provide a brief overview of the methodology used in the meta-analysis of CFRs in Figure **??**. We followed a standard methodology for systematic reviews, and all analyses were performed using the `meta` R package [2]. A mixed effects model is a linear model such that $y_i = \beta_0 + \sum_j \beta_j x_i + u_i + \epsilon_i$, where $\beta = (\beta_0, ..., \beta_j)^\top$ represent the fixed effects, y_i the observed data, x_i any explanatory variable and u_i are the random effect terms, centred around zero and independent across i, and ϵ_i are error terms. Meta-analysis is a special case of the above mixed-effects model with only an intercept term (β_0) and a random-effects term u_i associated with that intercept.

We derived a CFR for each study, either using the CFR directly as reported in the paper or by calculating the unadjusted CFR from outbreak data (deaths/cases). We then transformed the CFRs using logit-transformation $y_i = \log\left(\frac{CFR_i}{1-CFR_i}\right)$ to ensure that the distribution was approximately normal. Finally, we used a generalised logistic mixed-effects model with the transformed CFRs as the outcome to estimate the pooled effect.

A comprehensive overview of the methodology is provided by [3].

A.5 Quality Assessment

Possible responses for each question listed below were Yes, No, and Not applicable.

**Theme**	**Question**
Is the methodological/statistical approach suitable? (how the data are used)	1. Clear and reproducible
	2. Robust and appropriate for the aim [subjective criteria]
Are the assumptions appropriate? (input parameters/assumptions - what goes into the methodology)	3. Clear and reproducible
	4. Justified (published study or analysis of data)[objective criteria]
Are the data appropriate for the selected methodological approach?	5. Clearly described and reproducible
	6. Are issues in data clearly discussed and acknowledged?
	7. Are issues in data accounted for in chosen methodological approach?

Table A.1: Quality assessment questionnaire

B Additional Figures & Tables

**Inclusion**	**Exclusion**
Measures/estimates of human: Reproduction numbers (R, R_0, R_t, r, R_e), growth rate (r), doubling times, generation time, serial interval, incubation/latent period, case fatality ratio (CFR), attack rate, mutation rate (e.g. from phylogenetic study), overdispersion, risk factors (risk and the measure).	Non-English language publication
Mention of historical or any outbreak in humans: size, year, location, duration, spatial scale	Studies of co-infections. (local, regional, national, international).
Measures/estimates of animal: R, R_0, R_t, r, R_e, growth rate, mutation rate.	Animal studies that do not report R, R_t etc.
Mathematical or statistical model of transmission.	Qualitative studies, e.g., KAP studies.
Measures of seroprevalence and negative seroprevalence in humans.	Pathogen not the primary focus of study.
Relative ratio of human-human vs animal introductions.	Duplicates.
Reviews that report inclusion criteria for reference checking.	Does not match any of the inclusion criteria.
For "small" pathogens, include case reports to potentially reconstruct serial interval distribution etc.	In-vitro studies.
	Non-peer reviewed publications, conference proceedings, abstracts, posters, letters to the editor
	Papers that reference the city of Marburg in Germany instead of MVD

Table B.2: Inclusion and exclusion criteria.

Data field	Expected data type	Variable name	Notes
Outbreak ID	integer	outbreak_id	ID assigned by database
Article ID	integer	article_id	ID to connect to article form
Outbreak start day	integer	outbreak_start_day	Day of outbreak start if reported
Outbreak start month	character	outbreak_start_month	Month of outbreak start if reported
Outbreak start year	integer	outbreak_start_year	Year of outbreak start if reported
Outbreak end day	integer	outbreak_end_day	Day of outbreak end if reported
Outbreak end month	character	outbreak_end_month	Month of outbreak end if reported
Outbreak end year	integer	outbreak_date_year	Year of outbreak end if reported
Duration (months)	integer	outbreak_duration_months	Duration of outbreak in months, if reported. No calculation of duration is done.
Asymptomatic transmission described	logical	asymptomatic_transmission	Tick box whether asymptomatic transmission is described in the paper or not.
Outbreak country	character	outbreak_country	Country or countries where the outbreak took place - from dropdown list
Outbreak location	character	outbreak_location	Region/district/province/city where the outbreak took place
Cases confirmed	integer	cases_confirmed	Number of confirmed cases as reported
Mode of detection of cases	character	cases_mode_detection	Method for case detection - from dropdown list
Cases suspected	integer	cases_suspected	Number of suspected cases as reported
Asymptomatic cases	integer	cases_asymptomatic	Number of asymptomatic cases as reported
Deaths	integer	deaths	Number of deaths as reported

Table B.3: Outbreak form fields. Refer to epireview in Supplement C for dropdown options.

Data field	Expected data type	Variable name	Notes
Model data ID	integer	model_data_id	ID assigned by database
Article ID	integer	article_id	ID to connect to article form
Model type	character	model_type	General type of model - from dropdown list
Compartmental type	character	compartmental_type	Specific type of compartmental model - from dropdown list
Stochastic or deterministic	character	stoch_deter	Stochastic or deterministic model as reported
Theoretical model	logical	theoretical_model	Tick box whether the model was fitted to data (NA) or just theoretical (TRUE)
Intervention type	character	interventions_type	Type of intervention(s) modelled - from dropdown list
Code available	logical	code_available	Tick box whether code for model was publicly available and reported in the paper
Transmission route	character	transmission_route	Transmission route(s) modelled - from dropdown list
Assumptions	character	assumptions	General assumptions for the model - from dropdown list

Table B.4: Model form fields. Refer to epireview in Supplement C for dropdown options.

Data field	Expected data type	Variable name	Notes
Parameter data ID	integer	parameter_data_id	ID assigned by database
Article ID	integer	article_id	ID to connect to article form
Parameter type	character	parameter_type	Category of parameter - see dropdown list
Parameter value	numeric	parameter_value	Central parameter value
Parameter unit	character	parameter_unit	Units for parameter value, applies to central estimate and ranges/uncertainty intervals - see dropdown list
Parameter lower bound	numeric	parameter_lower_bound	Lower bound of the parameter range if a range was reported or if data are disaggregated
Parameter upper bound	numeric	parameter_upper_bound	Upper bound of the parameter range if a range was reported or if data are disaggregated
Parameter value type	character	parameter_value_type	Type of central parameter value - see dropdown list
Parameter uncertainty - single value	numeric	parameter_uncertainty_single_value	Value for uncertainty for central parameter value if a single value was reported (e.g. value of std. dev.)
Parameter uncertainty - single type	character	parameter_uncertainty_singe_type	Type of uncertainty fpr central parameter value if single value was reported - see dropdown list
Parameter uncertainty - lower value	numeric	parameter_uncertainty_lower_value	Lower bound for uncertainty for central parameter value if paired values were reported
Parameter uncertainty - upper value	numeric	parameter_uncertainty_upper_value	Upper bound for uncertainty for central parameter value if paired values were reported
Parameter uncertainty paired type	character	parameter_uncertainty_type	Type of uncertainty for central parameter value if paired values were reported - see dropdown list
Numerator	integer	cfr_ifr_numerator	Numerator of either CFR/IFR (deaths) or seroprevalence (number seropositive) estimates
Denominator	integer	cfr_ifr_denominator	Denominator of either CFR/IFR (cases) or seroprevalence (number tested) estimates
Distribution type	logical	distribution_type	Type of distribution for estimated parameter - see dropdown list

First distribution parameter value	logical	distribution_par1_value	Value for first distribution parameter (e.g. shape or scale parameter for a gamma distribution)
First distribution parameter type	logical	distribution_par1_type	Type of value for first distribution parameter - see dropdown list
First distribution parameter uncertainty	logical	distribution_par1_uncertainty	Tick box for whether uncertainty is estimated for the first distribution parameter (TRUE) or not (FALSE)
Second distribution parameter value	logical	distribution_par2_value	Value for second distribution parameter (e.g. shape or scale parameter for a gamma distribution)
Second distribution parameter type	logical	distribution_par2_type	Type of value for second distribution parameter - see dropdown list
Second distribution parameter uncertainty	logical	distribution_par2_uncertainty	Tick box for whether uncertainty is estimated for the second distribution parameter (TRUE) or not (FALSE)
Is parameter from supplement?	logical	method_from_supplement	Tick box for whether parameter was extracted from supplement (TRUE) or not (FALSE)
Survey timing related to outbreak	character	method_moment_value	Timing of the survey in relation to the outbreak, if specified in paper - see dropdown list
Is the CFR/IFR estimate adjusted?	character	cfr_ifr_method	Is the CFR/IFR estimate adjusted, unadjusted, or unspecified - see dropdown list
Method to estimate R	character	method_r	Method used for estimation of the reproduction number - see dropdown list
Parameter estimates disaggregated by	character	method_disaggregated_by	Categories for disaggregation of parameter estimates
Disaggregated data available	logical	method_disaggregated	Tick box if disaggregated estimates are available (TRUE) or not (FALSE)
Only disaggregated data available	logical	method_disaggregated_only	Tick box if ONLY disaggregated estimates are available (TRUE) or if a central estimate is also available (FALSE)
Outcome for risk factor(s)	character	riskfactor_outcome	Outcome for risk factor(s) - see dropdown list
Risk factor name	character	riskfactor_name	Risk factor name - see dropdown list
Risk factor occupation	character	riskfactor_occupation	If risk factor is an occupation, then specified occupation as risk factor - see dropdown list
Risk factor significant	character	riskfactor_significant	Statistical significance of risk factor(s) - see dropdown list
Risk factor adjusted	character	riskfactor_adjusted	Adjustment status of risk factor(s)- see dropdown list
Sex of study population	character	population_sex	Sex of survey population - see dropdown list
Population sample setting	character	population_sample_type	General setting of the survey - see dropdown list
Population group	character	population_group	Specific group of the survey population - see dropdown list
Study population minimum age (years)	numeric	population_age_min	Minimum age of the survey population in years
Study population maximum age (years)	numeric	population_age_max	Maximum age of the survey population in years
Study population sample size	integer	population_sample_size	Sample size of the population used for parameter estimation
Study population country	character	population_country	Country of the survey population - see dropdown list
Study population location	character	population_location	Region/district/province/city of the survey population - see dropdown list
Start day of study	integer	population_study_start_day	Study start day
Start month of study	character	population_study_start_month	Study start month - see dropdown list
Start year of study	integer	population_study_start_year	Study start year - see dropdown list
End day of study	integer	population_study_end_day	Study end day
End month of study	character	population_study_end_month	Study end month - see dropdown list
End year of study	integer	population_study_end_year	Study end year - see dropdown list
Genome site	character	genome_site	Site of genome or gene studied
Genomic sequence available?	logical	genomic_sequence_available	Tick box whether genomic sequence data are available (TRUE) or not (FALSE)

Parameter class	character	parameter_class	General parameter class (delays, seroprevalence, reproduction numbers, mutations, severity, risk factors, relative contribution)
Uncertainty	character	Uncertainty	Formatted uncertainty range from 'parameter_uncertainty_lower_value' and 'parameter_uncertainty_lower_value' in format x - x for ranges and x, x for confidence or credible intervals
Survey year	character	Survey year	Dates of survey in format YYYY, YYYY-YYYY, MMM YYYY, or MMM-MMM YYYY from survey start and end variables

Table B.5: Parameter form fields. Refer to epireview in Supplement C for dropdown options.

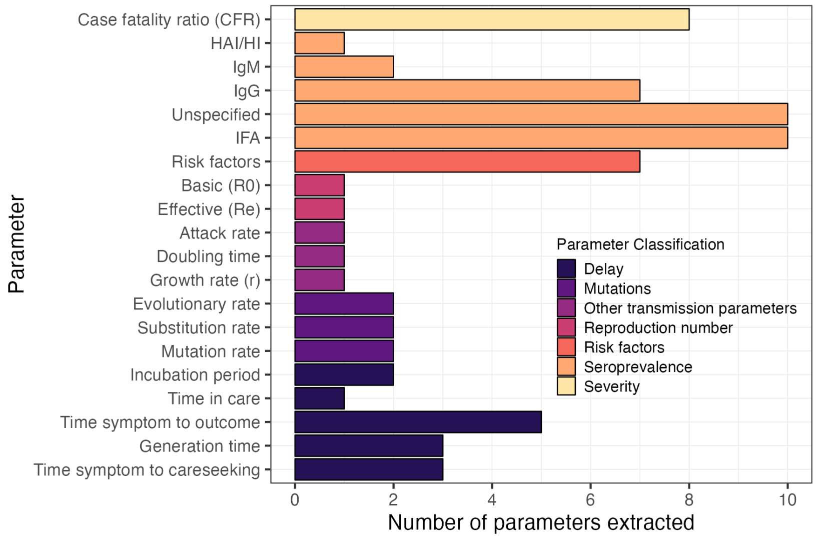

Figure B.1: The number of each type of parameter extracted from studies included in the review.

Article	Country	Survey year	Delay (days)	Statistic	Uncertainty (days)	Uncertainty type	Population Group	Timing of survey	Outcome
Generation Time									
Ajelli 2012 #	Angola	Mar-Nov 2005	9.3	Mean	3.7 - 14.6	CI95%	General population	Mid outbreak	
Ajelli 2012	Angola	Mar-Nov 2005	5.4	Standard Deviation	3.9 - 8.6	CI95%	General population	Mid outbreak	
Ajelli 2012 #	Angola	Mar-Nov 2005	9	Mean	8.2 - 10	CI95%	General population	Mid outbreak	
Incubation Period									
Martini 1973	Germany, Yugoslavia	Aug-Nov 1968			4 - 7	Range			
Gear 1975	South Africa	Feb 1975			7 - 8	Range	Healthcare workers	Mid outbreak	
Time In Care									
Knust 2015	Uganda	2012	14.3	Mean	4 - 22	Range	General population	Post outbreak	Other
Time From Symptom To Careseeking									
Bausch 2006	DRC*	1999-2000	4.5	Median	0 - 24	Range	Other	Mid outbreak	
Gear 1975	South Africa	Feb 1975	4	Other			Other	Start outbreak	
Knust 2015	Uganda	2012	4	Mean			General population		
Time From Symptom To Outcome									
Ajelli 2012	Angola	Mar-Nov 2005	7	Median	5 - 9	Range	General population	Mid outbreak	Death
Ajelli 2012	Angola	Mar-Nov 2005	9	Median	0 - 56	Range	General population	Mid outbreak	Death
Gear 1975	South Africa	Feb 1975	9				Other	Start outbreak	Other
Knust 2015	Uganda	2012	9	Mean	6.5 - 9	Range	General population	Post outbreak	Death
Knust 2015	Uganda	2012	22	Mean	16 - 30	Range	General population		Other

*DRC: Democratic Republic of the Congo
#Ajelli 2012 fits a model for this parameter with two different sets of assumptions.

Table B.6: Overview of the MVD delay parameter estimates extracted from the included studies. These are stratified into five categories: Generation Time, Incubation Period, Time in Care, Time from Symptom to Careseeking and Time from Symptom to Outcome. Estimates and associated uncertainty are provided, along with information regarding the population group corresponding to the estimate and the timing and location of the outbreak. 'Other' refers to a range of different values which are specified in the underlying papers.

Risk factor	Adjusted	Sample size (Significant)	Sample size (Not significant)
Infection			
Contact with animal	Unknown	Unknown	
Gathering	Unknown	128	
Household contact	Unknown	102	
Occupation - Funeral and burial services	Unknown	102	
Other	Unknown	102	26
Sex	Unknown		26
Seropositivity			
Contact with animal	Adjusted		912
Contact with animal	Unknown		300
Gathering	Unknown		300
Hospitalisation	Adjusted	915	
Household contact	Adjusted		912
Occupation - Funeral and burial services	Adjusted		912

Table B.7: Aggregated information on risk factors associated with MVD infection and seropositivity. Risk factors were mapped onto our risk factor classification (see Supplement) by interpreting the authors' descriptions. Adjusted refers to whether estimates were adjusted (i.e. from a multivariate analysis) or not (i.e. from a univariate analysis), with unknown showing that this information is not clearly stated in the original study. Statistical significance was determined according to the original authors' statistical approaches when specified, or using a p-value of 0.05 otherwise. The numbers in the significant and not significant columns represent the total sample size included in the analyses for this risk factor and outcome category.

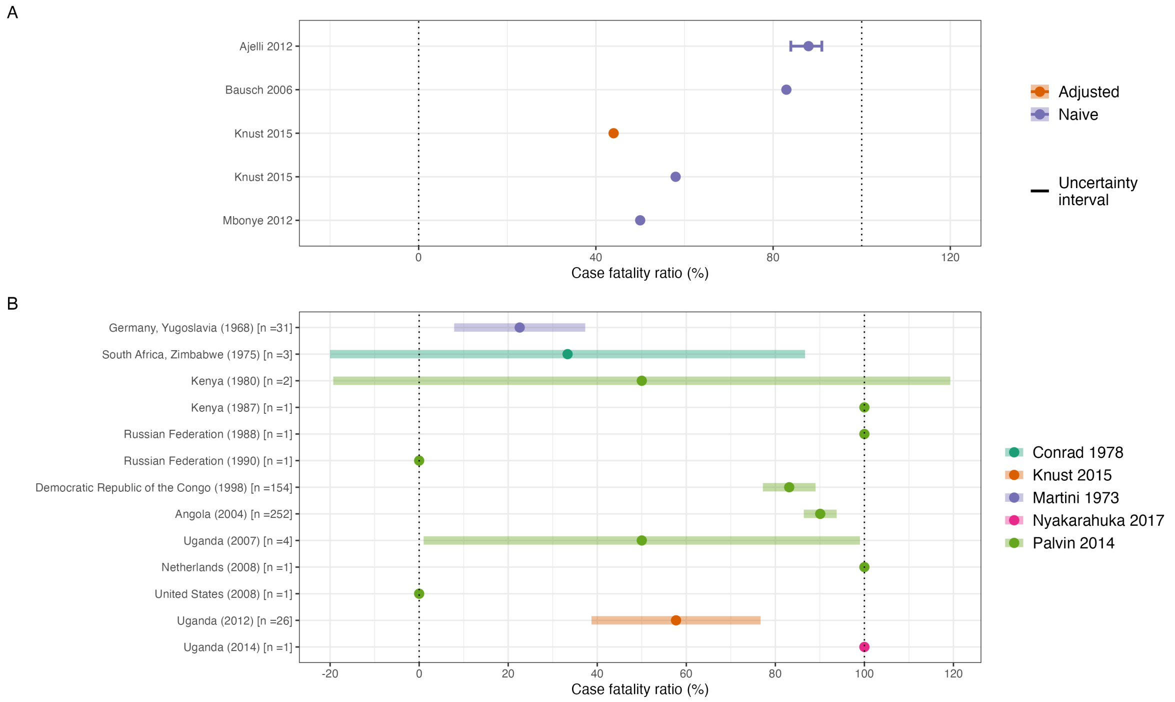

Figure B.2: Overview of the estimates of the case fatality ratio (CFR) obtained from the included studies. (A) CFR estimates reported in the included studies, stratified according to estimation method. Points represent central estimates. Error bars represent an uncertainty interval associated with the point estimate, as reported in the original study. (B) CFR estimated from extracted outbreak data, including only one observation per outbreak using the study with the longest duration of the outbreak reported ensuring each case is not double counted. Shaded bars represents the imputed binomial confidence interval for studies with a sample size, $n > 1$. Vertical dotted lines represent 0% and 100% CFR.

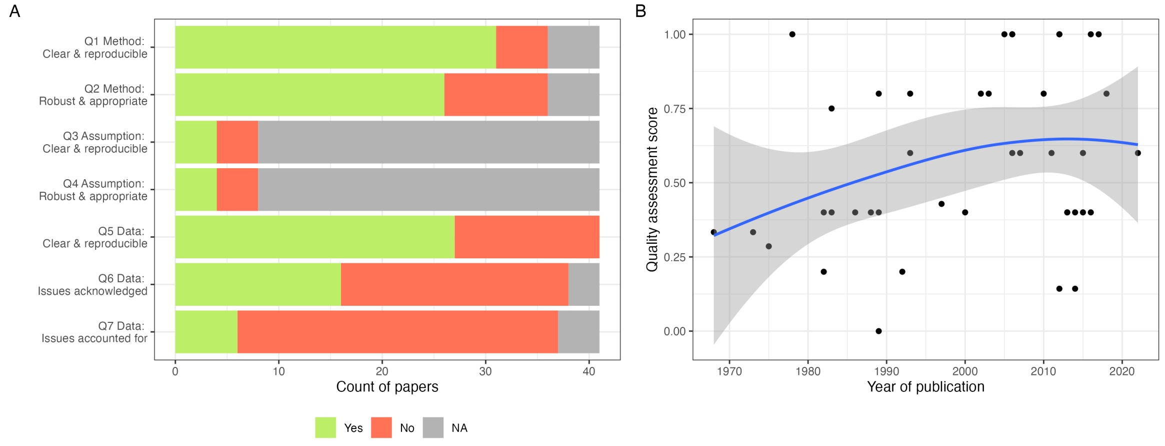

Figure B.3: (A) Count of papers for each quality assessment question scoring Yes, No or not applicable. (B) Quality Assessment Score defined as proportion of Yes votes for each paper relative to sum of Yes and No answers, removing NAs. The time trend is fitted using Local Polynomial Regression Fitting.

Article	Country	Survey year	Outcome	Risk factor	Significant	Adjusted	Sample size	Population sample type	Population group	Timing of survey
Borchert 2005	Angola	Mar-Jul 2005	Infection	Gathering	Significant	Unknown	102	Hospital based	Persons under investigation	Mid outbreak
Borchert 2005	Angola	Mar-Jul 2005	Infection	Occupation - Funeral and burial services	Significant	Unknown	102	Hospital based	Persons under investigation	Mid outbreak
Borchert 2005	Angola	Mar-Jul 2005	Infection	Household contact	Significant	Unknown	102	Hospital based	Persons under investigation	Mid outbreak
Borchert 2005	Angola	Mar-Jul 2005	Infection	Other	Significant	Unknown	102	Hospital based	Persons under investigation	Mid outbreak
Knust 2015	Uganda	2012	Infection	Gathering	Significant	Unknown	26	Community based	General population	Post outbreak
Knust 2015	Uganda	2012	Infection	Other	Not significant	Unknown	26	Community based	General population	Post outbreak
Knust 2015	Uganda	2012	Infection	Sex	Not significant	Unknown	26	Community based	General population	Post outbreak
Amman 2012	Uganda		Infection	Contact with animal	Significant	Unknown				
Amman 2012	Uganda		Infection	Other	Significant	Unknown				
Bausch 2003	Democratic Republic of the Congo	May 1999	Serology	Hospitalisation	Significant	Adjusted	915	Population based		
Bausch 2003	Democratic Republic of the Congo	May 1999	Serology	Contact with animal	Not significant	Adjusted	912	Population based		
Bausch 2003	Democratic Republic of the Congo	May 1999	Serology	Household contact	Not significant	Adjusted	912	Population based		
Bausch 2003	Democratic Republic of the Congo	May 1999	Serology	Occupation - Funeral and burial services	Not significant	Adjusted	912	Population based		
Borchert 2005	Democratic Republic of the Congo		Serology	Contact with animal	Not significant	Unknown	300	Community based		Post outbreak
Borchert 2005	Democratic Republic of the Congo		Serology	Gathering	Not significant	Unknown	300	Community based		Post outbreak

Figure B.4: More detailed table of risk factor data from the extracted studies, giving countries, times and contexts of surveys and non-aggregated information on each risk factor assessed in the four relevant studies.

C epireview

We developed an R package called *epireview* that provides a central location to host and access the extracted data for the nine priority pathogens, allows for submissions of outbreak, model, and parameter data from new peer-reviewed papers via pull requests, and includes functions to produce the figures and tables included in this paper and update them with any additional data. This package will be updated as the overall project by the Pathogen Epidemiology Review Group (PERG) continues to extract modelling parameters for the rest of the nine priority pathogens as defined by WHO.

There are several vignettes available:

- A vignette for MVD with tables and figures from this paper that will be updated as data are added to the database.
- A vignette that lists the options for each model, outbreak, or parameter field and describes how to access them using a function in the package.
- A vignette to explain the process of updating the database with new article, model, outbreak, or pathogen data.

D PRISMA 2020 Checklists

Section & Topic	Item #	Checklist item	Reported (Yes/No)
**Title**			
Title	1	Identify the report as a systematic review.	Yes
**Background**			
Objectives	2	Provide an explicit statement of the main objective(s) or question(s) the review addresses.	Yes
**Methods**			
Eligibility criteria	3	Specify the inclusion and exclusion criteria for the review.	Yes
Information sources	4	Specify the information sources (e.g. databases, registers) used to identify studies and the date when each was last searched.	Yes
Risk of bias	5	Specify the methods used to assess risk of bias in the included studies.	Yes
Synthesis of results	6	Specify the methods used to present and synthesise results.	Yes
**Results**			
Included studies	7	Give the total number of included studies and participants and summarise relevant characteristics of studies.	Yes
Synthesis of results	8	Present results for main outcomes, preferably indicating the number of included studies and participants for each. If meta-analysis was done, report the summary estimate and confidence/credible interval. If comparing groups, indicate the direction of the effect (i.e. which group is favoured).	Yes
**Discussion**			
Limitations of evidence	9	Provide a brief summary of the limitations of the evidence included in the review (e.g. study risk of bias, inconsistency and imprecision).	Yes
Interpretation	10	Provide a general interpretation of the results and important implications.	Yes
**Other**			
Funding	11	Specify the primary source of funding for the review.	Yes
Registration	12	Provide the register name and registration number.	Yes

Table D.8: PRISMA 2020 Abstracts Checklist. ([4])

Section & Topic	Item #	Checklist item	Location where item is reported
**Title**			
Title	1	Identify the report as a systematic review.	page 1
**Abstract**			
Abstract	2	See the PRISMA 2020 for Abstracts checklist.	Table D.8
**Introduction**			
Rationale	3	Describe the rationale for the review in the context of existing knowledge.	page 1
Objectives	4	Provide an explicit statement of the objective(s) or question(s) the review addresses.	page 1
**Methods**			
Eligibility criteria	5	Specify the inclusion and exclusion criteria for the review and how studies were grouped for the syntheses.	page 2
Information sources	6	Specify all databases, registers, websites, organisations, reference lists and other sources searched or consulted to identify studies. Specify the date when each source was last searched or consulted.	page 2
Search strategy	7	Present the full search strategies for all databases, registers and websites, including any filters and limits used.	page 2 + Figure 1

Table D.9: PRISMA 2020 Checklist. ([4])

Section & Topic	Item #	Checklist item	Location where item is reported
Selection process	8	Specify the methods used to decide whether a study met the inclusion criteria of the review, including how many reviewers screened each record and each report retrieved, whether they worked independently, and if applicable, details of automation tools used in the process.	page 2
Data collection process	9	Specify the methods used to collect data from reports, including how many reviewers collected data from each report, whether they worked independently, any processes for obtaining or confirming data from study investigators, and if applicable, details of automation tools used in the process.	page 2
Data items	10a	List and define all outcomes for which data were sought. Specify whether all results that were compatible with each outcome domain in each study were sought (e.g. for all measures, time points, analyses), and if not, the methods used to decide which results to collect.	page 2
	10b	List and define all other variables for which data were sought (e.g. participant and intervention characteristics, funding sources). Describe any assumptions made about any missing or unclear information.	page 2
Study risk of bias assessment	11	Specify the methods used to assess risk of bias in the included studies, including details of the tool(s) used, how many reviewers assessed each study and whether they worked independently, and if applicable, details of automation tools used in the process.	page 2
Effect measures	12	Specify for each outcome the effect measure(s) (e.g. risk ratio, mean difference) used in the synthesis or presentation of results.	page 2/3
Synthesis methods	13a	Describe the processes used to decide which studies were eligible for each synthesis (e.g. tabulating the study intervention characteristics and comparing against the planned groups for each synthesis (item 5)).	page 2/3
	13b	Describe any methods required to prepare the data for presentation or synthesis, such as handling of missing summary statistics, or data conversions.	page 2/3
	13c	Describe any methods used to tabulate or visually display results of individual studies and syntheses.	page 2/3
	13d	Describe any methods used to synthesize results and provide a rationale for the choice(s). If meta-analysis was performed, describe the model(s), method(s) to identify the presence and extent of statistical heterogeneity, and software package(s) used.	page 3
	13e	Describe any methods used to explore possible causes of heterogeneity among study results (e.g. subgroup analysis, meta-regression).	-
	13f	Describe any sensitivity analyses conducted to assess robustness of the synthesized results.	-
Reporting bias assessment	14	Describe any methods used to assess risk of bias due to missing results in a synthesis (arising from reporting biases).	page 2
Certainty assessment	15	Describe any methods used to assess certainty (or confidence) in the body of evidence for an outcome.	page 2
**Results**			
Study selection	16a	Describe the results of the search and selection process, from the number of records identified in the search to the number of studies included in the review, ideally using a flow diagram.	page 3
	16b	Cite studies that might appear to meet the inclusion criteria, but which were excluded, and explain why they were excluded.	Table F.11
Study characteristics	17	Cite each included study and present its characteristics.	pages 3-11
Risk of bias in studies	18	Present assessments of risk of bias for each included study.	page 6 + Figure B.3
Results of individual studies	19	For all outcomes, present, for each study: (a) summary statistics for each group (where appropriate) and (b) an effect estimate and its precision (e.g. confidence/credible interval), ideally using structured tables or plots.	pages 3-6
Results of syntheses	20a	For each synthesis, briefly summarise the characteristics and risk of bias among contributing studies.	page 5/6
	20b	Present results of all statistical syntheses conducted. If meta-analysis was done, present for each the summary estimate and its precision (e.g. confidence/credible interval) and measures of statistical heterogeneity. If comparing groups, describe the direction of the effect.	page 5
	20c	Present results of all investigations of possible causes of heterogeneity among study results.	page 5
	20d	Present results of all sensitivity analyses conducted to assess the robustness of the synthesized results.	page 5/6

Table D.9: PRISMA 2020 Checklist. ([4])

Section & Topic	Item #	Checklist item	Location where item is reported
Reporting biases	21	Present assessments of risk of bias due to missing results (arising from reporting biases) for each synthesis assessed.	page 6
Certainty of evidence	22	Present assessments of certainty (or confidence) in the body of evidence for each outcome assessed.	pages 3-6
**Discussion**			
Discussion	23a	Provide a general interpretation of the results in the context of other evidence.	pages 6-9
	23b	Discuss any limitations of the evidence included in the review.	page 8/9
	23c	Discuss any limitations of the review processes used.	page 8/9
	23d	Discuss implications of the results for practice, policy, and future research.	pages 6-9
**Other Information**			
Registration and protocol	24a	Provide registration information for the review, including register name and registration number, or state that the review was not registered.	page 9
	24b	Indicate where the review protocol can be accessed, or state that a protocol was not prepared.	page 9
	24c	Describe and explain any amendments to information provided at registration or in the protocol.	n/a
Support	25	Describe sources of financial or non-financial support for the review, and the role of the funders or sponsors in the review.	page 9
Competing interests	26	Declare any competing interests of review authors.	page 9
Availability of data, code and other materials	27	Report which of the following are publicly available and where they can be found: template data collection forms; data extracted from included studies; data used for all analyses; analytic code; any other materials used in the review.	page 9

Table D.9: PRISMA 2020 Checklist. ([4])

E Systematic Reviews

Systematic reviews were excluded from our analysis, but Table E.10 presents the systematic reviews which we have found and used for validation purposes.

Study	Title	Journal	DOI
Selvaraj 2018	Infection Rates and Risk Factors for Infection Among Health Workers During Ebola and Marburg Virus Outbreaks: A Systematic Review	The Journal of infectious diseases	10.1093/infdis/jiy435
Brainard 2016	Presence and Persistence of Ebola or Marburg Virus in Patients and Survivors: A Rapid Systematic Review	PLoS neglected tropical diseases	10.1371/journal.pntd.0004475
Brainard 2016	Risk factors for transmission of Ebola or Marburg virus disease: a systematic review and meta-analysis	International journal of epidemiology	10.1093/ije/dyv307
Tahmo 2023	An epidemiological synthesis of emerging and re-emerging zoonotic disease threats in Cameroon, 2000-2022: a systematic review	IJID Reg	10.1016/j.ijregi.2022.12.001
Nyakarahuka 2016	How severe and prevalent are Ebola and Marburg viruses? A systematic review and meta-analysis of the case fatality rates and seroprevalence	BMC infectious diseases	10.1186/s12879-016-2045-6

Table E.10: Excluded systematic reviews

F Excluded Studies

We list all excluded studies with reasons for exclusion in Table F.11

Study	Title	Journal	Notes
Zeller 2000	Infections by viruses of the families Bunyaviridae and Filoviridae	Revue scientifique et technique (International Office of Epizootics)	Exclusion reason: Reports metrics from other papers (not original estimates of primary data); JS (2019-03-21 21:30:42)(Screen): mention of historical outbreaks;
Wei 2017	Deep-sequencing of Marburg virus genome during sequential mouse passaging and cell-culture adaptation reveals extensive changes over time	Scientific reports	Exclusion reason: No report of parameters (including seroprevalence and other measures of interest) or transmission models or historical outbreaks; JS (2019-08-08 23:45:59)(Select): Yes, maybe someone with knowledge of genomics could calculate a mutation rate out of info here..?; KC (2019-03-19 23:05:13)(Screen): mutation rate?;
VanKerkhove 2015	A review of epidemiological parameters from Ebola outbreaks to inform early public health decision-making	Scientific data	Exclusion reason: Reports metrics from other papers (not original estimates of primary data);
Tukei 1996	Threat of Marburg and Ebola viral haemorrhagic fevers in Africa	East African medical journal	Exclusion reason: Reports metrics from other papers (not original estimates of primary data);
Sweileh 2017	Global research trends of World Health Organization's top eight emerging pathogens	Globalization and health	Exclusion reason: Wrong pathogen or pathogen epidemiology or transmission not main focus; JS (2019-08-08 19:54:12)(Select): Nothing super relevant transmission-wise though; JS (2019-08-08 19:53:58)(Select): Could check if we have similar split of disease papers as them; JS (2019-03-20 21:48:23)(Screen): massive review - good for reference checking;
Swanepoel 2007	Studies of reservoir hosts for Marburg virus	Emerging infectious diseases	Exclusion reason: No report of parameters (including seroprevalence and other measures of interest) or transmission models or historical outbreaks; JS (2019-08-08 19:49:49)(Select): Can we get mutation rate from the phylogenetic tree?; JS (2019-03-20 21:46:18)(Screen): mention of outbreak in humans;
Strickland-Cholmley 1970	Marburg virus	Lancet (London, England)	Exclusion reason: No report of parameters (including seroprevalence and other measures of interest) or transmission models or historical outbreaks;
Storm 2018	Antibody Responses to Marburg Virus in Egyptian Rousette Bats and Their Role in Protection against Infection	Viruses	Exclusion reason: No report of parameters (including seroprevalence and other measures of interest) or transmission models or historical outbreaks; JS (2019-08-08 19:41:03)(Select): There was some idea of infectious time, but not clearly outlined. Therefore not adding in ; JS (2019-03-13 04:22:07)(Screen): May contain data that could be used to estimate length of animal infectious period;
Spence 1982	Marburg virus disease–an indicator case in South Africa	South African medical journal = Suid-Afrikaanse tydskrif vir geneeskunde	Exclusion reason: No report of parameters (including seroprevalence and other measures of interest) or transmission models or historical outbreaks; JS (2019-08-09 18:39:34)(Select): correspondence, not a paper;
Snowden 1979	Marburg disease: the 20th Century	Zimbabwe Rhodesia Nurse	Exclusion reason: Full text not found;
Slenczka 2007	Forty years of marburg virus	The Journal of infectious diseases	Exclusion reason: Duplicate;

Table F.11: Excluded studies at full text review with exclusion reason

Study	Title	Journal	Notes
Slenczka 1999	The Marburg virus outbreak of 1967 and subsequent episodes	Current topics in microbiology and immunology	Exclusion reason: Reports metrics from other papers (not original estimates of primary data); JS (2019-08-23 18:53:35)(Select): Disease duration data (Table 4) - not sure what paper this comes from, also a table on secondary cases (possibly for attack rate - Table 3); JS (2019-03-20 21:24:52)(Screen): References Marburg outbreaks;
Slenczka 2017	Filovirus Research: How it Began	Current topics in microbiology and immunology	Exclusion reason: Not peer-reviewed paper; JS (2019-07-09 21:22:21)(Select): Book chapter; JS (2019-03-20 21:24:30)(Screen): mention of outbreak;
Siya 2019	Lowland grazing and Marburg virus disease (MVD) outbreak in Kween district, Eastern Uganda	BMC public health	Exclusion reason: No report of parameters (including seroprevalence and other measures of interest) or transmission models or historical outbreaks; JS (2019-08-07 23:38:17)(Select): collected opinions from people, no numbers;
Selvaraj 2018	Infection Rates and Risk Factors for Infection Among Health Workers During Ebola and Marburg Virus Outbreaks: A Systematic Review	The Journal of infectious diseases	Exclusion reason: Reports metrics from other papers (not original estimates of primary data); JS (2019-08-07 23:18:44)(Select): Has papers on risk factors;
Saluzzo 1981	[Antibodies against the Marburg virus among human populations in the southeastern Central African Republic]	Comptes rendus des seances de l'Academie des sciences. Serie III, Sciences de la vie	Exclusion reason: Not in English;
Rougeron 2015	Ebola and Marburg haemorrhagic fever	Journal of clinical virology : the official publication of the Pan American Society for Clinical Virology	Exclusion reason: Reports metrics from other papers (not original estimates of primary data); JS (2019-08-07 23:04:17)(Select): Has a list of outbreaks, but not much else; JS (2019-03-20 20:50:36)(Screen): Review - may have useful references;
Raabea 2012	Infection control during filoviral hemorrhagic Fever outbreaks	Journal of global infectious diseases	Exclusion reason: No report of parameters (including seroprevalence and other measures of interest) or transmission models or historical outbreaks;
Polonsky 2014	Emerging filoviral disease in Uganda: proposed explanations and research directions	The American journal of tropical medicine and hygiene	Exclusion reason: No report of parameters (including seroprevalence and other measures of interest) or transmission models or historical outbreaks; JS (2019-08-05 22:56:51)(Select): About Marburg but does not report parameters of interest; JS (2019-07-09 21:04:42)(Select): Perspectives; JS (2019-03-20 19:41:20)(Screen): mentions outbreaks, may contain useful references for the outbreaks;
Pittalis 2009	Case definition for Ebola and Marburg haemorrhagic fevers: a complex challenge for epidemiologists and clinicians	The new microbiologica	Exclusion reason: No report of parameters (including seroprevalence and other measures of interest) or transmission models or historical outbreaks; JS (2019-08-05 22:38:13)(Select): The table with case definition will help fill out the summary table, they have summarised what was considered a case in many outbreaks ; JS (2019-03-20 19:31:08)(Screen): may have data on incubation periods and so on;
Pigott 2015	Mapping the zoonotic niche of Marburg virus disease in Africa	Transactions of the Royal Society of Tropical Medicine and Hygiene	Exclusion reason: No report of parameters (including seroprevalence and other measures of interest) or transmission models or historical outbreaks; JS (2019-08-02 21:08:04)(Select): Not sure if this model is exactly what we want though; JS (2019-03-20 03:02:08)(Screen): modelling transmission;

Table F.11: Excluded studies at full text review with exclusion reason

Study	Title	Journal	Notes
Peterson 2016	Geographic potential of disease caused by Ebola and Marburg viruses in Africa	Acta tropica	Exclusion reason: No report of parameters (including seroprevalence and other measures of interest) or transmission models or historical outbreaks; JS (2019-08-01 23:21:41)(Select): Nevermind, the whole paper is about reservoir distribution, not really transmission. Therefore, the pathogen epidemiology and transmission aren't really a focus here.; JS (2019-03-20 19:27:36)(Screen): model of disease transmission;
Peterson 2006	Geographic potential for outbreaks of Marburg hemorrhagic fever	The American journal of tropical medicine and hygiene	Exclusion reason: No report of parameters (including seroprevalence and other measures of interest) or transmission models or historical outbreaks;
Peterson 2004	Potential mammalian filovirus reservoirs	Emerging infectious diseases	Exclusion reason: No report of parameters (including seroprevalence and other measures of interest) or transmission models or historical outbreaks; JS (2019-07-09 21:01:22)(Select): Perspective;
Peterson 2004	Ecologic and geographic distribution of filovirus disease	Emerging infectious diseases	Exclusion reason: No report of parameters (including seroprevalence and other measures of interest) or transmission models or historical outbreaks; JS (2019-08-01 01:25:58)(Select): Not really transmission modelling though - more like ecologic niche modelling; JS (2019-08-01 01:18:14)(Select): Has a map of the geographic distribution of Marburg; JS (2019-03-20 19:23:45)(Screen): possibly considers transmission in the model, if not, gives an estimate of the range of occurrence of Marburg which could be interesting for considering where the outbreaks have taken place;
Vella 1978	Lassa fever (LF) and Marburg disease (MVD): occurrences, origins and diagnoses	Royal Society of Health journal	Exclusion reason: No report of parameters (including seroprevalence and other measures of interest) or transmission models or historical outbreaks; JS (2019-03-21 20:19:00)(Screen): might be relevant (occurrences =outbreaks?) though paper will be hard to find;
Nyakarahuka 2017	Knowledge and attitude towards Ebola and Marburg virus diseases in Uganda using quantitative and participatory epidemiology techniques	PLoS neglected tropical diseases	Exclusion reason: No report of parameters (including seroprevalence and other measures of interest) or transmission models or historical outbreaks;
Ndayimirije 2005	Marburg hemorrhagic fever in Angola–fighting fear and a lethal pathogen	The New England journal of medicine	Exclusion reason: Not peer-reviewed paper; JS (2019-07-31 21:30:23)(Select): Also, no references.; JS (2019-07-09 20:47:19)(Select): Perspective piece - should be excluded?;
Natesan 2016	Human Survivors of Disease Outbreaks Caused by Ebola or Marburg Virus Exhibit Cross-Reactive and Long-Lived Antibody Responses	Clinical and vaccine immunology : CVI	Exclusion reason: No report of parameters (including seroprevalence and other measures of interest) or transmission models or historical outbreaks; JS (2019-03-19 23:23:35)(Screen): might be an interesting paper regarding duration of infectiousness;
Miraglia 2019	Marburgviruses: An Update	Laboratory medicine	Exclusion reason: Reports metrics from other papers (not original estimates of primary data); JS (2019-03-19 22:51:05)(Screen): Possibly useful review paper;
Mayega 2013	A descriptive overview of the burden, distribution and characteristics of epidemics in Uganda	East African journal of public health	Exclusion reason: Full text not found; JS (2019-12-12 00:17:22)(Select): Application rejected by supplier; JS (2019-10-18 02:13:35)(Select): Requested from library 17/10/2019;

Table F.11: Excluded studies at full text review with exclusion reason

Study	Title	Journal	Notes
Mahanty 2004	Pathogenesis of filoviral haemorrhagic fevers	The Lancet. Infectious diseases	Exclusion reason: Wrong pathogen or pathogen epidemiology or transmission not main focus; JS (2019-07-31 00:43:23)(Select): This paper is mostly about the cellular level response to Marburg, only thing of interest is that outbreak table but we would be getting that same table from more relevant reviews anyways;
MacNeil 2012	Ebola and Marburg hemorrhagic fevers: neglected tropical diseases?	PLoS neglected tropical diseases	Exclusion reason: Reports metrics from other papers (not original estimates of primary data);
Roddy 2007	The Medecins Sans Frontieres intervention in the Marburg hemorrhagic fever epidemic, Uige, Angola, 2005. II. lessons learned in the community	The Journal of infectious diseases	Exclusion reason: Wrong pathogen or pathogen epidemiology or transmission not main focus; JS (2019-08-07 21:16:33)(Select): Has an incubation period though they do not say where they get it from; JS (2019-03-20 19:57:44)(Screen): mention of outbreak;
Ligon 2005	Outbreak of Marburg hemorrhagic fever in Angola: a review of the history of the disease and its biological aspects	Seminars in pediatric infectious diseases	Exclusion reason: Reports metrics from other papers (not original estimates of primary data);
Leroy 2011	Ebola and Marburg haemorrhagic fever viruses: major scientific advances, but a relatively minor public health threat for Africa	Clinical microbiology and infection : the official publication of the European Society of Clinical Microbiology and Infectious Diseases	Exclusion reason: Reports metrics from other papers (not original estimates of primary data); JS (2019-03-19 21:26:24)(Screen): references outbreaks;
Leffel 2004	Marburg and Ebola viruses as aerosol threats	Biosecurity and bioterrorism : biodefense strategy, practice, and science	Exclusion reason: Reports metrics from other papers (not original estimates of primary data);
LeDuc 1989	Epidemiology of hemorrhagic fever viruses	Reviews of infectious diseases	Exclusion reason: No report of parameters (including seroprevalence and other measures of interest) or transmission models or historical outbreaks;
Lawrence 2005	Largest ever Marburg haemorrhagic fever outbreak, Angola	Euro surveillance : bulletin Europeen sur les maladies transmissibles = European communicable disease bulletin	Exclusion reason: Case report or case study (i.e. reports on less than 10 cases, but this threshold can be pathogen-dependent);
Kuzmin 2010	Marburg virus in fruit bat, Kenya	Emerging infectious diseases	Exclusion reason: No report of parameters (including seroprevalence and other measures of interest) or transmission models or historical outbreaks; JS (2019-07-09 18:24:16)(Select): Letter to Editor; JS (2019-03-19 21:20:04)(Screen): seroprevalence in bats?;
Kuroda 2014	A polymorphism of the TIM-1 IgV domain: implications for the susceptibility to filovirus infection	Biochemical and biophysical research communications	Exclusion reason: No report of parameters (including seroprevalence and other measures of interest) or transmission models or historical outbreaks; JS (2019-03-13 04:13:23)(Screen): Mentions a mortality rate so this paper may contain a reference to a paper that has CFR ;
Kortepeter 2011	Basic clinical and laboratory features of filoviral hemorrhagic fever	The Journal of infectious diseases	Exclusion reason: Reports metrics from other papers (not original estimates of primary data); JS (2019-07-30 19:06:07)(Select): references for incubation period, CFR;
Klenk 2017	Marburg- and Ebolaviruses: A Look Back and Lessons for the Future	Methods in molecular biology (Clifton, N.J.)	Exclusion reason: Not peer-reviewed paper; JS (2019-07-09 18:15:39)(Select): Book chapter; JS (2019-03-19 20:50:51)(Screen): has a list of Marburg outbreaks;

Table F.11: Excluded studies at full text review with exclusion reason

Study	Title	Journal	Notes
"Kalter 1969	Antibodies in primates to the Marburg virus	Proceedings of the Society for Experimental Biology and Medicine. Society for Experimental Biology and Medicine (New York, N.Y.)	Exclusion reason: No report of parameters (including seroprevalence and other measures of interest) or transmission models or historical outbreaks; JS (2019-08-23 18:46:38)(Select): looks like negative seroprevalence in humans; JS (2019-07-09 18:10:20)(Select): Available from ICL library; JS (2019-03-13 04:10:26)(Screen): Can possibly involve humans - humans are primates ;
" Hayman 2015	Biannual birth pulses allow filoviruses to persist in bat populations	Proceedings. Biological sciences	Exclusion reason: No report of parameters (including seroprevalence and other measures of interest) or transmission models or historical outbreaks; JS (2019-03-15 00:11:50)(Screen): a mathematical model of bat-human transmission?;
Hartman 2010	Ebola and marburg hemorrhagic fever	Clinics in laboratory medicine	Exclusion reason: Reports metrics from other papers (not original estimates of primary data);
Grolla 2011	The use of a mobile laboratory unit in support of patient management and epidemiological surveillance during the 2005 Marburg Outbreak in Angola	PLoS neglected tropical diseases	Exclusion reason: Wrong pathogen or pathogen epidemiology or transmission not main focus; JS (2019-07-30 00:48:51)(Select): More about the performance of the laboratory than epidemiology and transmission;
Green 2012	Uganda battles Marburg fever outbreak	Lancet	Exclusion reason: Case report or case study (i.e. reports on less than 10 cases, but this threshold can be pathogen-dependent);
Glaze 2015	A Comparison of the Pathogenesis of Marburg Virus Disease in Humans and Nonhuman Primates and Evaluation of the Suitability of These Animal Models for Predicting Clinical Efficacy under the 'Animal Rule'	Comparative medicine	Exclusion reason: Reports metrics from other papers (not original estimates of primary data); JS (2019-07-22 23:27:49)(Select): This review has summarised Marburg outbreaks and has lots of references; JS (2019-03-19 00:55:16)(Screen): Review may have details on R0 etc.;
Gilsdorf 2012	Guidance for contact tracing of cases of Lassa fever, Ebola or Marburg haemorrhagic fever on an airplane: results of a European expert consultation	BMC public health	Exclusion reason: Reports metrics from other papers (not original estimates of primary data); JS (2019-07-22 23:22:18)(Select): mentions incubation periods and historical outbreaks, and has a handy table;
Geisbert 2015	Considerations in the Use of Nonhuman Primate Models of Ebola Virus and Marburg Virus Infection	The Journal of infectious diseases	Exclusion reason: Reports metrics from other papers (not original estimates of primary data); JS (2019-03-19 03:09:08)(Screen): Mentions case-fatality rates in humans - probably not the original source paper though;
Gear 1975	Outbreake of Marburg virus disease in Johannesburg	British medical journal	Exclusion reason: Duplicate;
Gear 1989	Clinical aspects of African viral hemorrhagic fevers	Reviews of infectious diseases	Exclusion reason: Reports metrics from other papers (not original estimates of primary data); JS (2019-07-22 21:31:03)(Select): does not report any parameters of interest of Marburg and we have the original paper on the Jburg outbreak;
Gear 1982	The hemorrhagic fevers of Southern Africa with special reference to studies in the South African Institute for Medical Research	The Yale journal of biology and medicine	Exclusion reason: Reports metrics from other papers (not original estimates of primary data); JS (2019-07-22 19:45:30)(Select): Review-type article but no references and not much Marburg;

Table F.11: Excluded studies at full text review with exclusion reason

Study	Title	Journal	Notes
Galbraith 1980	Changing patterns of communicable disease in England and Wales. Part i–Newly recognised diseases	British medical journal	Exclusion reason: Reports metrics from other papers (not original estimates of primary data); JS (2019-07-22 19:02:16)(Select): Doesn't have references for Marburg? Also, does not deal with Marburg on its own in a meaningful way;
Fisher-Hoch 2005	Lessons from nosocomial viral haemorrhagic fever outbreaks	British medical bulletin	Exclusion reason: Wrong pathogen or pathogen epidemiology or transmission not main focus; JS (2019-07-19 22:54:00)(Select): Not really about Marburg on its own;
Fernando 2015	Immune Response to Marburg Virus Angola Infection in Nonhuman Primates	The Journal of infectious diseases	Exclusion reason: No report of parameters (including seroprevalence and other measures of interest) or transmission models or historical outbreaks; JS (2019-03-18 20:56:47)(Screen): Historical outbreak mentioned + CFR mentioned only in abstract/intro, not the point of the study;
Feldmann 1996	Emerging and reemerging of filoviruses	Archives of virology. Supplementum	Exclusion reason: Duplicate;
Feldmann 1996	Marburg and Ebola viruses	Advances in virus research	Exclusion reason: Reports metrics from other papers (not original estimates of primary data); JS (2019-08-14 22:25:32)(Select): A pretty old review, nothing here that the newer reviews would not cover. I think we can safely exclude.;
Feldmann 1996	Filoviruses	Medical Microbiology	Exclusion reason: Not peer-reviewed paper; JS (2019-06-27 22:54:08)(Select): Book chapter;
Ewers 2016	Natural History of Aerosol Exposure with Marburg Virus in Rhesus Macaques	Viruses	Exclusion reason: Wrong pathogen or pathogen epidemiology or transmission not main focus; PD (2023-03-14 02:23:55)(Select): Moved back to exclusion because it's an animal challenge trial; JS (2019-03-18 20:48:01)(Screen): Incubation period in animals - we care about this for animals, right? But at the same time it is an experimental study...;
Enserink 2005	Infectious diseases. A puzzling outbreak of Marburg disease	Science	Exclusion reason: Not peer-reviewed paper; JS (2019-06-27 22:43:23)(Select): news - not peer-reviewed;
Emanuel 2018	Filoviruses: Ecology, Molecular Biology, and Evolution	Advances in virus research	Exclusion reason: Not peer-reviewed paper; JS (2019-06-27 22:39:31)(Select): Book chapter - not a paper;
Dowdle 1976	Marburg virus	Bulletin of the Pan American Health Organization	Exclusion reason: Reports metrics from other papers (not original estimates of primary data);
Dimitrov 2008	Adaptive modeling of viral diseases in bats with a focus on rabies	Journal of theoretical biology	Exclusion reason: Wrong pathogen or pathogen epidemiology or transmission not main focus; JS (2019-07-18 19:05:20)(Select): The model is about bat rabies, not Marburg; JS (2019-03-15 21:56:23)(Screen): Spillover modelling;
Curtis 2006	Viral haemorrhagic fevers caused by Lassa, Ebola and Marburg viruses	Advances in experimental medicine and biology	Exclusion reason: Not peer-reviewed paper; JS (2019-06-27 22:32:11)(Select): Conference paper;
Johnson 1996	Characterization of a new Marburg virus isolated from a 1987 fatal case in Kenya	Archives of virology. Supplementum	Exclusion reason: Not peer-reviewed paper; JS (2019-08-09 18:41:51)(Select): exclude, not a peer-reviewed paper; JS (2019-06-28 00:28:18)(Select): conference paper; JS (2019-03-19 20:19:33)(Screen): case study;
Johnson 1982	Marburg, Ebola and Rift Valley Fever virus antibodies in East African primates	Transactions of the Royal Society of Tropical Medicine and Hygiene	Exclusion reason: Wrong pathogen or pathogen epidemiology or transmission not main focus; JS (2019-08-23 20:56:56)(Select): Has seroprevalence amongst human animal handlers; JS (2019-07-30 00:38:12)(Select): animal handlers were all Marburg -; JS (2019-03-19 20:10:14)(Screen): seroprevalence in animals;

Table F.11: Excluded studies at full text review with exclusion reason

Study	Title	Journal	Notes
Jeffs 2007	The Medecins Sans Frontieres intervention in the Marburg hemorrhagic fever epidemic, Uige, Angola, 2005. I. Lessons learned in the hospital	The Journal of infectious diseases	Exclusion reason: No report of parameters (including seroprevalence and other measures of interest) or transmission models or historical outbreaks;
Jeffs 2006	A clinical guide to viral haemorrhagic fevers: Ebola, Marburg and Lassa	Tropical doctor	Exclusion reason: Wrong pathogen or pathogen epidemiology or transmission not main focus; JS (2019-07-29 21:12:02)(Select): Deals with Ebola and Marburg together, gives both same incubation period etc. Not really Marburg-specific. Marking as wrong pathogen; JS (2019-03-19 20:08:52)(Screen): may have info on CFR, incubation period etc.;
"Isaacson 2001	Viral hemorrhagic fever hazards for travelers in Africa	Clinical infectious diseases : an official publication of the Infectious Diseases Society of America	Exclusion reason: Wrong pathogen or pathogen epidemiology or transmission not main focus; JS (2019-07-29 21:00:17)(Select): Deals with Marburg and Ebola together, not very helpful. Tagged as wrong pathogen etc. because of the joining of the two ;
" Colebunders 2007	Marburg hemorrhagic fever in Durba and Watsa, Democratic Republic of the Congo: clinical documentation, features of illness, and treatment	The Journal of infectious diseases	Exclusion reason: Reports metrics from other papers (not original estimates of primary data); JS (2019-07-18 19:03:52)(Select): CFR split by whether or not patients received healthcare; JS (2019-07-18 19:02:36)(Select): Onset to symptoms;
Colebunders 2004	Organisation of health care during an outbreak of Marburg haemorrhagic fever in the Democratic Republic of Congo, 1999	The Journal of infection	Exclusion reason: No report of parameters (including seroprevalence and other measures of interest) or transmission models or historical outbreaks; JS (2019-07-18 18:51:33)(Select): The outbreak in this paper is at least partially covered by other papers (eg. the one with the infant by Borchert et al.). I think that this paper does not really have new info about the transmission of that outbreak and they do not provide risk factors. I'll include for now just in case we don't have all the references in this paper in the review list. ;
Changula 2014	Ebola and Marburg virus diseases in Africa: increased risk of outbreaks in previously unaffected areas?	Microbiology and immunology	Exclusion reason: Reports metrics from other papers (not original estimates of primary data); JS (2019-03-15 21:07:35)(Screen): Mention of outbreaks;
Callendret 2018	A prophylactic multivalent vaccine against different filovirus species is immunogenic and provides protection from lethal infections with Ebolavirus and Marburgvirus species in non-human primates	PloS one	Exclusion reason: No report of parameters (including seroprevalence and other measures of interest) or transmission models or historical outbreaks; JS (2019-03-19 00:53:30)(Screen): CFR in primates?;
Burton 2004	Marburg miner mystery	Lancet Infect Dis	Exclusion reason: Not peer-reviewed paper; JS (2019-06-27 22:16:07)(Select): news;
Brown 1997	Threat to Humans from Virus Infections of Non-human Primates	Reviews in medical virology	Exclusion reason: Wrong pathogen or pathogen epidemiology or transmission not main focus; JS (2019-07-18 00:09:51)(Select): Not worth keeping - everything here can be found elsewhere and Marburg not the main focus; JS (2019-03-15 20:53:38)(Screen): Mention of outbreak;

Table F.11: Excluded studies at full text review with exclusion reason

Study	Title	Journal	Notes
Brett-Major 2018	Catching Chances: The Movement to Be on the Ground and Research Ready before an Outbreak	Viruses	Exclusion reason: Wrong pathogen or pathogen epidemiology or transmission not main focus;
Brauburger 2012	Forty-five years of Marburg virus research	Viruses	Exclusion reason: Reports metrics from other papers (not original estimates of primary data);
”Bramble 2018	Pan-Filovirus Serum Neutralizing Antibodies in a Subset of Congolese Ebolavirus Infection Survivors	The Journal of infectious diseases	Exclusion reason: Wrong pathogen or pathogen epidemiology or transmission not main focus; JS (2019-07-17 23:49:42)(Select): This paper is about Ebola survivors, not Marburg infection ;
” Brainard 2016	Presence and Persistence of Ebola or Marburg Virus in Patients and Survivors: A Rapid Systematic Review	PLoS neglected tropical diseases	Exclusion reason: Wrong pathogen or pathogen epidemiology or transmission not main focus; JS (2019-07-17 23:47:32)(Select): Mostly about Ebola; JS (2019-03-15 20:21:18)(Screen): Review may contain useful references;
Brainard 2016	Risk factors for transmission of Ebola or Marburg virus disease: a systematic review and meta-analysis	International journal of epidemiology	Exclusion reason: Reports metrics from other papers (not original estimates of primary data); JS (2019-07-17 21:27:14)(Select): Risk factors possibly in the relevant references;
Borchert 2005	Lessons from the outbreak of Marburg virus	N Engl J Med	Exclusion reason: Not peer-reviewed paper;
Borchert 2000	Viewpoint: filovirus haemorrhagic fever outbreaks: much ado about nothing?	Tropical medicine	international health : TM
IH	Exclusion reason: No report of parameters (including seroprevalence and other measures of interest) or transmission models or historical outbreaks;		
Bonney 2013	Hospital-based surveillance for viral hemorrhagic fevers and hepatitides in Ghana	PLoS neglected tropical diseases	Exclusion reason: No report of parameters (including seroprevalence and other measures of interest) or transmission models or historical outbreaks; CM (2023-05-01 02:28:54)(Select): We have other sero-studies which report 0 prevalence ...; JS (2019-03-15 00:59:52)(Screen): Found a seroprevalence of 0 in humans;
”Bonn 2005	Marburg fever in Angola: still a mystery disease	Lancet Infect Dis	Exclusion reason: Not peer-reviewed paper; JS (2019-03-15 00:34:02)(Screen): Not really a journal article - more of an interview ;
” Beer 1999	Characteristics of Filoviridae: Marburg and Ebola viruses	Die Naturwissenschaften	Exclusion reason: Reports metrics from other papers (not original estimates of primary data); JS (2019-03-14 23:15:16)(Screen): Info on Marburg outbreaks and case fatality;
Bebell 2015	Ebola virus disease and Marburg disease in pregnancy: a review and management considerations for filovirus infection	Obstetrics and gynecology	Exclusion reason: Reports metrics from other papers (not original estimates of primary data); JS (2019-03-14 23:08:12)(Screen): Review;
Bausch 2008	Treatment of Marburg and Ebola hemorrhagic fevers: a strategy for testing new drugs and vaccines under outbreak conditions	Antiviral research	Exclusion reason: Reports metrics from other papers (not original estimates of primary data); JS (2019-03-15 00:05:01)(Screen): Case fatality rate mentioned - may have a reference for it;

Table F.11: Excluded studies at full text review with exclusion reason

Study	Title	Journal	Notes
Barrette 2011	Current perspectives on the phylogeny of Filoviridae	Infection, genetics and evolution : journal of molecular epidemiology and evolutionary genetics in infectious diseases	Exclusion reason: No report of parameters (including seroprevalence and other measures of interest) or transmission models or historical outbreaks; JS (2019-03-15 00:09:59)(Screen): Mentions outbreaks and Marburg - may have relevant references;
"Bannister 2010	Viral haemorrhagic fevers imported into non-endemic countries: risk assessment and management	British medical bulletin	Exclusion reason: Wrong pathogen or pathogen epidemiology or transmission not main focus; JS (2019-03-14 23:05:01)(Screen): Not solely about filoviruses ;
" Ascenzi 2008	Ebolavirus and Marburgvirus: insight the Filoviridae family	Molecular aspects of medicine	Exclusion reason: Reports metrics from other papers (not original estimates of primary data); JS (2019-03-14 22:27:02)(Screen): Looks like a review - may have useful references;
Amman 2014	Marburgvirus resurgence in Kitaka Mine bat population after extermination attempts, Uganda	Emerging infectious diseases	Exclusion reason: No report of parameters (including seroprevalence and other measures of interest) or transmission models or historical outbreaks;
Amman 2015	Oral shedding of Marburg virus in experimentally infected Egyptian fruit bats (Rousettus aegyptiacus)	Journal of wildlife diseases	Exclusion reason: No report of parameters (including seroprevalence and other measures of interest) or transmission models or historical outbreaks; JS (2019-03-14 22:18:34)(Screen): May contain human outbreak info;
"Alves 2010	Aerosol exposure to the angola strain of marburg virus causes lethal viral hemorrhagic Fever in cynomolgus macaques	Veterinary pathology	Exclusion reason: Wrong pathogen or pathogen epidemiology or transmission not main focus; JS (2019-03-14 21:55:37)(Screen): may get an animal estimate of incubation period ;
" Allaranga 2010	Lessons learned during active epidemiological surveillance of Ebola and Marburg viral hemorrhagic fever epidemics in Africa	East African journal of public health	Exclusion reason: Reports metrics from other papers (not original estimates of primary data); JS (2019-06-17 19:27:59)(Select): Contains list of Marburg outbreaks - though no original data. Good to retain for reference-checking?;
Alfson 2018	A Single Amino Acid Change in the Marburg Virus Glycoprotein Arises during Serial Cell Culture Passages and Attenuates the Virus in a Macaque Model of Disease	mSphere	Exclusion reason: No report of parameters (including seroprevalence and other measures of interest) or transmission models or historical outbreaks; JS (2019-03-13 04:05:06)(Screen): Can possibly calculate CFR in animal model from study data;
Adjemian 2011	Outbreak of Marburg hemorrhagic fever among miners in Kamwenge and Ibanda Districts, Uganda, 2007	The Journal of infectious diseases	Exclusion reason: Duplicate; JS (2019-03-13 03:48:12)(Screen): Duplicate; JS (2019-03-13 03:45:48)(Screen): Outbreak;
	Case definitions. Ebola-Marburg viral diseases	Epidemiological bulletin	Exclusion reason: Not peer-reviewed paper;
	After Marburg, Ebola	Lancet	Exclusion reason: Wrong pathogen or pathogen epidemiology or transmission not main focus; JS (2019-08-24 01:50:42)(Select): About Ebola;
Polonsky 2014	Perspective Piece Emerging Filoviral Disease in Uganda: Proposed Explanations and Research Directions	American Journal of Tropical Medicine and Hygiene	Exclusion reason: Duplicate; JS (2019-03-20 19:42:35)(Screen): duplicate;

Table F.11: Excluded studies at full text review with exclusion reason

Study	Title	Journal	Notes
Okeke 2014	Diagnostic schemes for reducing epidemic size of african viral hemorrhagic fever outbreaks	Journal of Infection in Developing Countries	Exclusion reason: Wrong pathogen or pathogen epidemiology or transmission not main focus; JS (2019-08-01 00:36:24)(Select): The model seems to be parameterised and used on only Ebola; JS (2019-03-20 02:44:44)(Screen): Focuses on Ebola but paper may also consider Marburg?;
Nyakarahuka 2014	Using network analysis technique to describe the spread of Marburg hemorrhagic fever outbreak in Uganda, 2012	International Journal of Infectious Diseases	Exclusion reason: Not peer-reviewed paper; JS (2019-07-09 20:50:31)(Select): Oral presentation;
Nakayama 2011	Ebola and Marburg viruses	Journal of Disaster Research	Exclusion reason: Reports metrics from other papers (not original estimates of primary data); JS (2019-03-19 23:17:23)(Screen): mention of outbreaks;
Kupradze 1981	VIRAL HEMORRHAGIC FEVERS	Izvestiya Akademii Nauk Gruzinskoi SSR Seriya Biologicheskaya	Exclusion reason: Not in English;
Kortepeter 2011	Basic Clinical and Laboratory Features of Filoviral Hemorrhagic Fever	Journal of Infectious Diseases	Exclusion reason: Duplicate; JS (2019-03-19 20:56:53)(Screen): duplicate;
Rodhain 1989	ARBOVIRUS INFECTIONS AND VIRAL HEMORRHAGIC FEVERS IN UGANDA - A SEROLOGICAL SURVEY IN KARAMOJA DISTRICT, 1984	Transactions of the Royal Society of Tropical Medicine and Hygiene	Exclusion reason: Duplicate; JS (2019-03-15 01:02:03)(Screen): Strictly speaking not Marburg alone, but useful for serological data;
Roddy 2007	The medecins sans frontieres intervention in the Marburg hemorrhagic fever epidemic, Uige, Angola, 2005. II. lessons learned in the community	Journal of Infectious Diseases	Exclusion reason: Duplicate; JS (2019-03-20 19:58:19)(Screen): duplicate;
Roddy 2009	Decreased peripheral health service utilisation during an outbreak of Marburg haemorrhagic fever, Uige, Angola, 2005	Transactions of the Royal Society of Tropical Medicine and Hygiene	Exclusion reason: Reports metrics from other papers (not original estimates of primary data); JS (2019-08-05 23:35:41)(Select): Not really about Marburg in itself;
Knust 2015	Multidistrict Outbreak of Marburg Virus Disease-Uganda, 2012	Journal of Infectious Diseases	Exclusion reason: Duplicate; JS (2019-03-19 20:51:53)(Screen): duplicate;
"Kalter 1971	A SEROLOGICAL SURVEY OF PRIMATE SERA FOR ANTIBODY TO THE MARBURG VIRUS	Martini, G. a. and R. Siegert	Exclusion reason: Not peer-reviewed paper; JS (2019-07-09 18:08:11)(Select): Book chapter; JS (2019-03-13 04:08:23)(Screen): Also contains seroprevalence data in humans - humans are primates ;
" Johnson 1996	Characterization of a new Marburg virus isolated from a 1987 fatal case in Kenya	Archives of Virology Supplement	Exclusion reason: Duplicate; JS (2019-03-19 20:19:15)(Screen): duplicate;

Table F.11: Excluded studies at full text review with exclusion reason

Study	Title	Journal	Notes
Johnson 1993	HEMORRHAGIC-FEVER VIRUS ACTIVITY IN EQUATORIAL AFRICA - DISTRIBUTION AND PREVALENCE OF FILOVIRUS REACTIVE ANTIBODY IN THE CENTRAL-AFRICAN-REPUBLIC	Transactions of the Royal Society of Tropical Medicine and Hygiene	Exclusion reason: Duplicate; JS (2019-03-19 20:11:34)(Screen): seroprevalence in humans;
Johnson 1983	VIRAL HEMORRHAGIC-FEVER SURVEILLANCE IN KENYA, 1980-1981	Tropical and Geographical Medicine	Exclusion reason: Duplicate; JS (2019-03-19 20:10:51)(Screen): duplicate;
Jeffs 2007	The medecins sans frontieres intervention in the Marburg hemorrhagic fever epidemic, Uige, Angola, 2005. I. lessons learned in the hospital	Journal of Infectious Diseases	Exclusion reason: Duplicate; JS (2019-03-19 20:09:17)(Screen): duplicate;
Ivanoff 1982	HEMORRHAGIC-FEVER IN GABON .1. INCIDENCE OF LASSA, EBOLA AND MARBURG VIRUSES IN HAUT-OGOOUE	Transactions of the Royal Society of Tropical Medicine and Hygiene	Exclusion reason: Duplicate; JS (2019-03-19 03:59:55)(Screen): seroprevalence?;
Gonzalez 1983	AFRICAN VIRAL HEMORRHAGIC FEVERS STUDIES IN THE CENTRAL-AFRICAN-REPUBLIC	Cahiers O.R.S.T.O.M. (Office de la Recherche Scientifique et Technique Outre-Mer) Serie Entomologie Medicale et Parasitologie	Exclusion reason: Not in English;
Towner 2007	High-throughput molecular detection of hemorrhagic fever virus threats with applications for outbreak settings	Journal of Infectious Diseases	Exclusion reason: No report of parameters (including seroprevalence and other measures of interest) or transmission models or historical outbreaks; JS (2019-08-08 23:02:40)(Select): Did analysis of 500 people for Uige outbreak – 180+/505; JS (2019-03-21 20:23:53)(Screen): may contain data on original seroprevalence in these 500 people;
Sureau 1989	Recent findings on the African viral haemorrhagic fevers	Maladies tropicales transmissibles.	Exclusion reason: Not in English; JS (2019-03-20 21:44:43)(Screen): may be in French?;
Slenczka 2007	Forty years of Marburg virus	Journal of Infectious Diseases	Exclusion reason: Reports metrics from other papers (not original estimates of primary data);
Martini 1971	MARBURG VIRUS DISEASE CLINICAL SYNDROME	Martini, G. a. and R. Siegert	Exclusion reason: Not peer-reviewed paper; JS (2019-07-09 18:35:24)(Select): Book chapter; KC (2019-03-19 22:44:35)(Screen): incubation period from first outbreak reported here;
Lub 1995	Clinical and virologic characterization of the disease in guinea pigs aerogenically infected with Marburg virus	Voprosy Virusologii	Exclusion reason: Not in English; JS (2019-03-19 21:46:23)(Screen): incubation period etc. in guinea pigs;
Ftika 2013	Viral haemorrhagic fevers in healthcare settings	Journal of Hospital Infection	Exclusion reason: Wrong pathogen or pathogen epidemiology or transmission not main focus; JS (2019-07-19 23:32:31)(Select): Review talks a lot about Ebola and Marburg together. Not really Marburg-specific;

Table F.11: Excluded studies at full text review with exclusion reason

Study	Title	Journal	Notes
Freitas 2017	Filovirus strains, the environment conditions and the bats in and out of Africa	Archives of Veterinary Science	Exclusion reason: No report of parameters (including seroprevalence and other measures of interest) or transmission models or historical outbreaks; JS (2019-03-18 23:14:01)(Screen): outbreak range;
Formenty 2006	Viral haemorrhagic fevers in the world: review of the last ten years	Bulletin Epidemiologique Hebdomadaire	Exclusion reason: Not in English; JS (2019-10-18 02:13:23)(Select): Requested from library 17/10/2019;
Ford 1999	Haemorrhagic fever in Democratic Republic of Congo identified as Marburg	Lancet	Exclusion reason: Not peer-reviewed paper; JS (2019-06-27 23:00:07)(Select): news;
Fernando 2015	Immune Response to Marburg Virus Angola Infection in Nonhuman Primates	Journal of Infectious Diseases	Exclusion reason: Duplicate;
Feldmann 2006	Focus on research: Marburg hemorrhagic fever - The forgotten cousin strikes	New England Journal of Medicine	Exclusion reason: Reports metrics from other papers (not original estimates of primary data); JS (2019-07-18 19:49:59)(Select): Perspective – I think we're excluding these;
Feldmann 1996	Emerging and reemerging of filoviruses	Archives of Virology	Exclusion reason: Not peer-reviewed paper; JS (2019-07-11 19:47:59)(Select): Book chapter;
Farnon 2009	FILOVIRUS SEROURVEY FOLLOWING AN OUTBREAK OF MARBURG HEMORRHAGIC FEVER — IBANDA AND KAMWENGE DISTRICTS, UGANDA, 2007	American Journal of Tropical Medicine and Hygiene	Exclusion reason: Not peer-reviewed paper; JS (2019-08-09 18:40:33)(Select): conference proceedings - exclude;
Falzarano 2006	Characterization of Marburg virus from a recent outbreak in Angola	American Journal of Tropical Medicine and Hygiene	Exclusion reason: Not peer-reviewed paper; JS (2019-08-09 18:40:17)(Select): conference proceedings - exclude;
Eichenlaub 1985	HEMORRHAGIC FEVERS RISK FOR HOSPITAL STAFF	Hygiene + Medizin	Exclusion reason: Not in English;
Dietrich 1978	Marburg virus disease	Ebola virus haemorrhagic fever. Proceedings of an international colloquium on Ebola virus infection and other haemorrhagic fevers held in Antwerp, Belgium, 6-8 December, 1977.	Exclusion reason: Not peer-reviewed paper; JS (2019-06-27 22:34:53)(Select): Proceedings of an International Colloquium;
Colebunders 2007	Marburg hemorrhagic fever in durba and watsa, democratic republic of the congo: Clinical documentation, features of illness, and treatment	Journal of Infectious Diseases	Exclusion reason: Duplicate;
Tessier 1987	VIRAL HEMORRHAGIC-FEVER SURVEY IN CHOBE (BOTSWANA)	Transactions of the Royal Society of Tropical Medicine and Hygiene	Exclusion reason: Wrong pathogen or pathogen epidemiology or transmission not main focus; JS (2019-08-08 20:00:16)(Select): zero prevalence of Marburg;
Hibbs 1993	Epidemic of febrile disease in Berbera, Somalia	Journal of Tropical Medicine	Exclusion reason: No report of parameters (including seroprevalence and other measures of interest) or transmission models or historical outbreaks; JS (2019-03-19 03:38:52)(Screen): seroprevalence in humans?;
Hennessen 1971	EPIDEMIOLOGY OF MARBURG VIRUS DISEASE	Martini, G. a. and R. Siegert	Exclusion reason: Not peer-reviewed paper; JS (2019-07-29 20:01:17)(Select): Book chapter?;

Table F.11: Excluded studies at full text review with exclusion reason

Study	Title	Journal	Notes
Henderson 1971	EPIDEMIOLOGICAL STUDIES IN UGANDA RELATING TO THE MARBURG AGENT	Martini, G. a. and R. Siegert	Exclusion reason: Not peer-reviewed paper; JS (2019-07-29 20:01:03)(Select): Book chapter?;
Chepurnova 2000	Assay of Marburg virus in the blood and secretions of experimentally infected animals	Voprosy Virusologii	Exclusion reason: Not in English; JS (2019-03-15 00:06:20)(Screen): Animal incubation period;
Cdc 2005	Brief report: Outbreak of Marburg virus hemorrhagic fever - Angola, October 1, 2004-March 29, 2005 (Reprinted from MMWR, vol 54, pg 308-309, 2005)	Jama-Journal of the American Medical Association	Exclusion reason: Not peer-reviewed paper;
Cardenas 2013	Marburg marburgvirus	Mononegaviruses of veterinary importance. Volume I: Pathobiology and molecular diagnosis	Exclusion reason: Not peer-reviewed paper; JS (2019-06-27 22:18:25)(Select): book chapter;
Buchmeier 1984	MARBURG AND EBOLA VIRUSES NEW AGENTS ON THE FRONTIERS OF VIROLOGY	Notkins, a. L. and M. B. a. Oldstone	Exclusion reason: Not peer-reviewed paper; JS (2019-06-27 22:19:14)(Select): book chapter; JS (2019-03-15 20:57:05)(Screen): Mention of historical outbreaks;
Belanov 1996	Survival of Marburg virus on contaminated surfaces and in aerosol	Voprosy Virusologii	Exclusion reason: Not in English; JS (2019-05-17 19:59:07)(Select): In Russian;
Bausch 1999	Investigation of an outbreak of Marburg hemorrhagic fever in the Democratic Republic of Congo	American Journal of Tropical Medicine and Hygiene	Exclusion reason: Not peer-reviewed paper; JS (2019-08-08 23:57:12)(Select): Conference proceedings - not a journal article; JS (2019-05-17 19:53:23)(Select): Could not find the paper;
Anonymous 2005	Outbreak of Marburg virus hemorrhagic fever - Angola, October 1, 2004-March 29, 2005	Morbidity and Mortality Weekly Report	Exclusion reason: Case report or case study (i.e. reports on less than 10 cases, but this threshold can be pathogen-dependent); JS (2019-06-26 23:54:47)(Select): This report is from the middle of the outbreak, so technically does not report parameters of interest;
Amman 2017	Ecology of Filoviruses	Marburg- and Ebolaviruses: from Ecosystems to Molecules	Exclusion reason: Not peer-reviewed paper; JS (2019-05-17 19:44:39)(Select): Book chapter;
Amdiouni 2015	Ebola virus and other Filoviruses: an overview	Journal of Coastal Life Medicine	Exclusion reason: Reports metrics from other papers (not original estimates of primary data); KC (2019-07-02 18:47:26)(Select): mostly Ebola; JS (2019-03-14 21:57:29)(Screen): About filoviruses - may contain a review of Marburg?;
Borchert 2002	A cluster of Marburg virus disease involving an infant	Tropical Medicine	International Health
Exclusion reason: Duplicate;			
Boardman 2003	Viral hemorrhagic fever	Primary Care Update for Ob/Gyns	Exclusion reason: Reports metrics from other papers (not original estimates of primary data); JS (2019-03-15 00:29:36)(Screen): incubation period estimate;
"Adegboro 2011	Marburg haemorrhagic fever: recent advances	African Journal of Clinical and Experimental Microbiology	Exclusion reason: No report of parameters (including seroprevalence and other measures of interest) or transmission models or historical outbreaks; JS (2019-03-13 03:40:08)(Screen): A review? ;
"	Outbreak news. Marburg haemorrhagic fever, Uganda	Releve epidemiologique hebdomadaire	Exclusion reason: Not peer-reviewed paper; JS (2019-03-20 23:58:32)(Screen): Not really a journal article but adding it in anyways because it's kind of a case;

Table F.11: Excluded studies at full text review with exclusion reason

Study	Title	Journal	Notes
	Largest marburg outbreak ever recorded hits Angola	Biosecurity and Bioterrorism-Biodefense Strategy Practice and Science	Exclusion reason: Not peer-reviewed paper; JS (2019-08-08 23:55:45)(Select): This seems to not really be a journal article, but a bunch of summaries? Probably not a peer-reviewed journal article?;
Adepoju 2021	West Africa on alert for haemorrhagic fevers	Lancet	Exclusion reason: Case report or case study (i.e. reports on less than 10 cases, but this threshold can be pathogen-dependent);
Araf 2023	Marburg virus outbreak in 2022: a public health concern	Lancet Microbe	Exclusion reason: No report of parameters (including seroprevalence and other measures of interest) or transmission models or historical outbreaks;
Asad 2020	Past and current advances in Marburg virus disease: a review	Infez Med	Exclusion reason: Reports metrics from other papers (not original estimates of primary data);
Baby 2022	Sagacious perceptive on Marburg virus foregrounding the recent findings :A Critical Review	Infect Disord Drug Targets	Exclusion reason: Reports metrics from other papers (not original estimates of primary data);
Bouba 2023	Predicting the combined effects of case isolation, safe funeral practices, and contact tracing during Ebola virus disease outbreaks	PLoS One	Exclusion reason: Wrong pathogen or pathogen epidemiology or transmission not main focus;
Chakraborty 2022	Sexual transmission of recently re-emerged deadly Marburg virus (MARV) needs explorative studies and due attention for its prevention and feasible spread - Correspondence	Int J Surg	Exclusion reason: No report of parameters (including seroprevalence and other measures of interest) or transmission models or historical outbreaks;
Filion 2023	Preliminary Investigation of Schmalhausen's Law in a Directly Transmitted Pathogen Outbreak System	Viruses	Exclusion reason: No report of parameters (including seroprevalence and other measures of interest) or transmission models or historical outbreaks;
Fujita-Fujiharu 2022	Structural insight into Marburg virus nucleoprotein-RNA complex formation	Nat Commun	Exclusion reason: No report of parameters (including seroprevalence and other measures of interest) or transmission models or historical outbreaks;
Harris 2023	WHO: Marburg Virus Outbreak Confirmed in Equatorial Guinea	Jama	Exclusion reason: Not peer-reviewed paper; CM (2023-05-01 19:43:17)(Select): Case report for Equatorial Guinea. Should we include?;
Hunter 2023	Marburg Fever	StatPearls	Exclusion reason: No report of parameters (including seroprevalence and other measures of interest) or transmission models or historical outbreaks;
Iannetta 2019	Viral Hemorrhagic Fevers Other than Ebola and Lassa	Infect Dis Clin North Am	Exclusion reason: Reports metrics from other papers (not original estimates of primary data);
Islam 2023	Epidemiology, pathophysiology, transmission, genomic structure, treatment, and future perspectives of the novel Marburg virus outbreak	Int J Surg	Exclusion reason: Case report or case study (i.e. reports on less than 10 cases, but this threshold can be pathogen-dependent);

Table F.11: Excluded studies at full text review with exclusion reason

Study	Title	Journal	Notes
Jacobs 2023	They come in threes: Marburg virus, emerging infectious diseases, and the blood supply	Transfus Apher Sci	Exclusion reason: Not peer-reviewed paper; CM (2023-05-01 19:38:43)(Select): This would only be recording the (recent) outbreak with 2 cases in Ghana in 2022;
Janik 2020	Dangerous Pathogens as a Potential Problem for Public Health	Medicina (Kaunas)	Exclusion reason: Wrong pathogen or pathogen epidemiology or transmission not main focus;
Jonkmans 2021	Scoping future outbreaks: a scoping review on the outbreak prediction of the WHO Blueprint list of priority diseases	BMJ Glob Health	Exclusion reason: No report of parameters (including seroprevalence and other measures of interest) or transmission models or historical outbreaks;
Koundouno 2022	Detection of Marburg Virus Disease in Guinea	N Engl J Med	Exclusion reason: Case report or case study (i.e. reports on less than 10 cases, but this threshold can be pathogen-dependent);
Languon 2019	Filovirus Disease Outbreaks: A Chronological Overview	Virology (Auckl)	Exclusion reason: No report of parameters (including seroprevalence and other measures of interest) or transmission models or historical outbreaks;
Mohapatra 2022	Recent re-emergence of Marburg virus disease in an African country Ghana after Guinea amid the ongoing COVID-19 pandemic: Another global threat? Current knowledge and strategies to tackle this highly deadly disease having feasible pandemic potential	Int J Surg	Exclusion reason: No report of parameters (including seroprevalence and other measures of interest) or transmission models or historical outbreaks;
Olejnik 2019	Recent advances in marburgvirus research	F1000Res	Exclusion reason: Reports metrics from other papers (not original estimates of primary data);
Ristanovifá 2020	A Forgotten Episode of Marburg Virus Disease: Belgrade, Yugoslavia, 1967	Microbiol Mol Biol Rev	Exclusion reason: Reports metrics from other papers (not original estimates of primary data); CM (2023-05-01 02:32:02)(Select): Includes parameter estimates from prior non-english studies;
Sah 2022	Marburg virus re-emerged in 2022: recently detected in Ghana, another zoonotic pathogen coming up amid rising cases of Monkeypox and ongoing COVID-19 pandemic- global health concerns and counteracting measures	Vet Q	Exclusion reason: No report of parameters (including seroprevalence and other measures of interest) or transmission models or historical outbreaks;
Sahoo 2023	The Marburg virus outbreak in West Africa	Curr Drug Targets	Exclusion reason: Not peer-reviewed paper;
Shifflett 2019	Marburg virus pathogenesis - differences and similarities in humans and animal models	Virol J	Exclusion reason: Reports metrics from other papers (not original estimates of primary data);
Stephens 2022	Drivers of African Filovirus (Ebola and Marburg) Outbreaks	Vector Borne Zoonotic Dis	Exclusion reason: No report of parameters (including seroprevalence and other measures of interest) or transmission models or historical outbreaks;

Table F.11: Excluded studies at full text review with exclusion reason

Study	Title	Journal	Notes
Tahmo 2023	An epidemiological synthesis of emerging and re-emerging zoonotic disease threats in Cameroon, 2000-2022: a systematic review	IJID Reg	Exclusion reason: No report of parameters (including seroprevalence and other measures of interest) or transmission models or historical outbreaks;
Woolsey 2020	Immune correlates of postexposure vaccine protection against Marburg virus	Sci Rep	Exclusion reason: No report of parameters (including seroprevalence and other measures of interest) or transmission models or historical outbreaks;
Mashkoor 2022	Recurrent Marburg virus disease outbreaks from 1967 to 2022: A perspective on challenges imposed and future implications	ASIAN PACIFIC JOURNAL OF TROPICAL MEDICINE	Exclusion reason: No report of parameters (including seroprevalence and other measures of interest) or transmission models or historical outbreaks;

Table F.11: Excluded studies at full text review with exclusion reason

G Pathogen Epidemiology Review Group (PERG) membership

First Name	Surname	Primary Affiliation
Aaron	Morris	University of Oxford
Alpha	Forna	University of Georgia
Amy	Dighe	Johns Hopkins
Anne	Cori	Imperial College London
Arran	Hamlet	Imperial College London
Ben	Lambert	Manchester University
Charlie	Whittaker	Imperial College London
Christian	Morgenstern	Imperial College London
Cyril	Geismar	Imperial College London
Dariya	Nikitin	Imperial College London
David	Jorgensen	Imperial College London
Ed	Knock	Imperial College London
Ettie	Unwin	Imperial College London
Gina	Cuomo-Dannenburg	Imperial College London
Hayley	Thompson	PATH
Isobel	Routledge	UCSF
Janetta	Skarp	Imperial College London
Joseph	Hicks	Imperial College London
Keith	Fraser	Imperial College London
Kelly	Charniga	Imperial College London
Kelly	McCain	Imperial College London
Lily	Geidelberg	Imperial College London
Lorenzo	Cattarino	UKHSA
Mara	Kont	Imperial College London
Marc	Baguelin	Imperial College London
Natsuko	Imai	Imperial College London
Nima	Moghaddas	Imperial College London
Patrick	Doohan	Imperial College London
Rebecca	Nash	Imperial College London
Ruth	McCabe	University of Oxford
Sabine	van Elsland	Imperial College London
Sangeeta	Bhatia	Imperial College London
Sreejith	Radhakrishnan	University of Glasgow
Zulma	Cucunuba Perez	Pontificia Universidad Javeriana
Jack	Wardle	Imperial College London

Table G.12: Pathogen Epidemiology Review Group (PERG) membership

References

1. Ajelli M and Merler S. Transmission potential and design of adequate control measures for Marburg hemorrhagic fever. PloS one 2012; 7:e50948. DOI: 10.1371/journal.pone.0050948. Available from: https://www.ncbi.nlm.nih.gov/pmc/articles/PMC3519495/pdf/pone.0050948.pdf
2. Balduzzi S, Rücker G, and Schwarzer G. How to perform a meta-analysis with R: a practical tutorial. Evidence-Based Mental Health 2019 :153–60
3. Harrer M, Cuijpers P, A FT, and Ebert DD. Doing Meta-Analysis With R: A Hands-On Guide. 1st. Boca Raton, FL and London: Chapman Hall/CRC Press, 2021
4. Page MJ, McKenzie JE, Bossuyt PM, Boutron I, Hoffmann TC, Mulrow CD, Shamseer L, Tetzlaff JM, Akl EA, Brennan SE, et al. The PRISMA 2020 statement: an updated guideline for reporting systematic reviews. International journal of surgery 2021; 88:105906
